# Selecting the foremost big data tool to optimize YouTube data in dynamic Fermatean fuzzy knowledge

**DOI:** 10.1371/journal.pone.0307381

**Published:** 2024-08-23

**Authors:** Dilshad Alghazzawi, Abdul Razaq, Hanan Alolaiyan, Aqsa Noor, Hamiden Abd El-Wahed Khalifa, Qin Xin

**Affiliations:** 1 Department of Mathematics, College of Science & Arts, King Abdulaziz University, Rabigh, Saudi Arabia; 2 Division of Science and Technology, Department of Mathematics, University of Education, Lahore, Pakistan; 3 Department of Mathematics, College of Science, King Saud University, Riyadh, Saudi Arabia; 4 Department of Mathematics, College of Science, Qassim University, Buraydah, Saudi Arabia; 5 Department of Operations and Management Research, Faculty of Graduate Studies for Statistical Research, Cairo University, Giza, Egypt; 6 Faculty of Science and Technology, University of the Faroe Islands, Faroe Islands, Denmark; Istanbul University: Istanbul Universitesi, TÜRKIYE

## Abstract

Big data pertains to extensive and intricate compilations of information that necessitate the implementation of proficient and cost-effective evaluation and analysis tools to derive insights and support decision making. The Fermatean fuzzy set theory possesses remarkable capability in capturing imprecision due to its capacity to accommodate complex and ambiguous problem descriptions. This paper presents the study of the concepts of dynamic ordered weighted aggregation operators in the context of Fermatean fuzzy environment. In numerous practical decision making scenarios, the term "dynamic" frequently denotes the capability of obtaining decision-relevant data at various time intervals. In this study, we introduce two novel aggregation operators: Fermatean fuzzy dynamic ordered weighted averaging and geometric operators. We investigate the attributes of these operators in detail, offering a comprehensive description of their salient features. We present a step-by-step mathematical algorithm for decision making scenarios in the context of proposed methodologies. In addition, we highlight the significance of these approaches by presenting the solution to the decision making problem and determining the most effective big data analytics platform for YouTube data analysis. Finally, we perform a thorough comparative analysis to assess the effectiveness of the suggested approaches in comparison to a variety of existing techniques.

## 1. Introduction

The principal aim of aggregation operators is to merge a variety of values into a unified output, guaranteeing that every value is thoroughly incorporated in the final aggregated outcome. Before the advent of aggregation operators, conventional crisp sets were widely utilized as decision-making frameworks. The complexity of decision-making is greatly increased by ambiguity and voids in information [1–3]. Problem-solving is significantly impeded by variables including uncertainty, imprecision, and insufficient data. Standard real numbers pose difficulties when confronted with ambiguous conditions [4, 5]. The theory of fuzzy sets (FS) was first proposed by Zadeh in 1965 [6]. Its exceptional achievements are attributable to its adeptness in uncertainty management. FS can be defined as indicating insufficiency or ambiguity in a given dataset solely through membership degrees. A major turning point in this domain transpired with the introduction of the theory of intuitionistic fuzzy sets (IFS) by Atanassov [7]. An IFS is distinguished by the presence of both membership and non-membership functions, as opposed to a FS which is solely defined by its membership function. The ability of the IFS to incorporate ambiguity introduced by data impression is a significant advantage. Consequently, IFS can be implemented in a variety of domains, particularly in the decision-making process.

An inherent obstacle that emerges during the process of decision-making is the synthesis of disparate information obtained from a multitude of sources. The incorporation of this integration is crucial for the decision-making process. To achieve precise aggregation, researchers have utilized myriad of methodology, including fusion-specific techniques, rule-based approaches, possibility theory, probability theory and FS theory. These strategies are dependent on distinctive quantitative aggregation operations, which are mathematical formulas that are essential for condensing a collection of data into a single, unique value. Numerous aggregation operations have been formulated to combine knowledge represented by IFS originating from different experts, alternatives, and temporal contexts. In 2007, seminal intuitionistic fuzzy aggregation operators were introduced [8], comprising IFWA, IFOWA, and IFHA. The article [9] deliberated on geometric operators such as IFWG, IFOWG, and IFHG within the framework of IF environment. These operators were further applied in the domain of MADM, specifically grounded in the principles of IFS. Li [10] performed a study on the application of generalized ordered weighted averaging operations to IF data. Induced geometric aggregation operators were first proposed in the context of IF information by the study reported in [11]. Since the inception of IFS in 1986, the incorporation of IFS has garnered substantial attention and has proven efficacious in addressing MADM problems. Garg [12] introduced updated forms of cosine similarity measures for IFS and utilized them in the decision-making process. In [13], Seikh and Mandal define Dombi aggregation operators under IF settings.

Notably, if the aggregate of membership and non-membership degrees exceeds 1, the operational definition of an IFS ceases to be feasible. As a consequence, the Pythagorean fuzzy set (PFS) was established in 2013 [14]. In the context of PFS, the total of the squares of the degrees of membership and non-membership is restricted to the interval [0,1]. PFS are capable of handling scenarios with higher levels of uncertainty compared to IFS. Therefore, PFS demonstrates greater effectiveness than IFS in tackling practical issues. The Pythagorean fuzzy weighted averaging and geometric operators were formulated in 2013 [15] under PFS, and employed to address MADM problems. In study [16] notion of symmetric PF weighted geometric/averaging operators was introduced. Zhang [17] presented the Pythagorean fuzzy ordered weighted averaging operator. Garg [18] developed various types of weighted averaging operators for Pythagorean fuzzy data and applied them to MADM problems. Rahman et al. [19] introduced the Pythagorean fuzzy ordered weighted geometric operator, elucidating its fundamental features and demonstrating its application to MADM problems. The PFS proves to outperform IFS in its capacity to accurately represent and analyze intricate ambiguity within practical decision-making scenarios. Although PFS expands the range of potential options, it is necessary to develop a more advanced version of fuzzy sets capable of addressing scenarios that fall beyond the scope of the PFS framework.

Senapati and Yager [20] proposed the concept of "Fermatean fuzzy sets" (FFSs), a conceptual framework that extends beyond the limitations of IFS and PFS. FFS have enhanced flexibility due to the constraint 0≤*α*^3^+*β*^3^≤1. This adaptability is particularly advantageous in situations involving decision-making that are characterized by ambiguity. Presently, FFS is recognized as the most extensively accepted collection of fuzzy sets in the field. In their work documented in [21], the scholars presented a novel set of operations tailored for FFSs, encompassing division, subtraction and Fermatean arithmetic mean operations, within the same scholarly contribution, they advocated the application of the Fermatean fuzzy weighted product model as a strategic approach to tackle MADM problems. In 2019, the notion of Fermatean fuzzy weighted averaging and geometric operators were proposed [22], wherein their practical applications in the realm of decision-making were extensively studied. The academic discussion on Fermatean fuzzy sets (FFSs) has quickly acquired popularity among many scholars, as seen by the significant attention they have received in a variety of research. For instant, selection of electric vehicle charging station [23] within FF framework, the authors in [24] introduced some score functions on FFS, the study [25] presented university’s recruitment process under FF knowledge, selection of waste disposal location [26] within FF environment, the authors in [27] have presented many FF capital budgeting approaches under FF knowledge. An investigation was undertaken in [28] regarding FFS and its utilization in the assessment and choice of cold chain logistics distribution centers. Garg [29] introduced an extended MABAC approach that incorporates prospect theory into the domain of FF knowledge. Kakati et al. [30] introduced the FF Archimedean Heronian model for transport under FF settings. Seikh et al. [31] presented Interval-valued FF Dombi aggregation operators within Interval-valued FF environment. In reference [32] the scholars introduced the interval-valued FF TOPSIS method under Interval-valued FF knowledge.

The study of aggregation operators is a substantial and fascinating research domain within the field of decision-making. Over recent decades, various operators have been proposed, particularly the ordered weighted averaging (OWA) [33] and ordered weighted geometric (OWG) [34] operators, which are generally recognized. The authors [35], introduced FF Dombi OWA and FF Dombi OWG in their study using the FF framework. Garg [36] proposed FFYOWA and FFYOWG operators as extensions to the FF environment. The solutions to diverse MADM problems that have been solved in various environments using aggregation operators can be found in the references [37–45].

### 1.1. Importance of FF theory

FFSs are better equipped to handle higher levels of uncertainty compared to IFSs and PFSs. FFSs are more suited for handling situations that include heightened uncertainty and complexity in MADM challenges. For instance, these sets can efficiently accommodate situations such as (0.9, 0.6), or (0.7, 0.8), where IFS and PFS criteria do not satisfy this situation due to the sum of membership and non-membership exceeding 1. On the other hand, FFS adeptly manages this situation under the stipulation that the cubic sum of its degrees of membership and non-membership do not surpass one, such that 0.9^3^+0.6^3^ = 0.94≤1. This situation demonstrates that FFS is considered more versatile and superior tool in MADM problems than IFS and PFS.

### 1.2. Significance of dynamic aggregation operators in the framework of FF environment

The aforementioned research endeavors predominantly focus on decision-making scenarios characterized by the simultaneous presentation of all initial decision data. Although in numerous decision-making contexts, such as dynamic assessment of big data analytics, dynamic assessment of military system efficiency, dynamic medical diagnostics, dynamic medical treatment, personnel dynamic evaluation, and multi-period investment decision-making, vital information required for making decisions must be gathered at varying time intervals. Time degrees are used in situations requiring dynamic IF numbers in the framework of MADM. A study on dynamic intuitionistic fuzzy MADM was conducted in [46], in which the authors proposed the dynamic IF aggregation operators. Wei, G. W. [47] introduced many geometric aggregation functions and demonstrated their application in dynamic MADM in an IF environment. In 2023, the notion of dynamic aggregation operators under complex Intuitionistic fuzzy knowledge was proposed in [48]. These dynamic aggregation operators were established for IFS that met the constraint that the sum of membership and non-membership degrees did not exceed 1. However, there are several scenarios in MADM where the aggregate of membership and non-membership degrees exceeds 1. This matter emphasizes the significance of establishing dynamic aggregation operators in the FF context.

The primary benefit of this study is that the FFS has a more extensive framework in comparison to the IFS, as it satisfies the requirement α^3+β^3≤1. Therefore, it is more appropriate for dealing with dynamic decision-making scenarios that entail uncertainty. Therefore, it is crucial to carry out research that addresses the resolution of problems associated with dynamic fuzzy MADM. This research investigates dynamic FF MADM, representing attribute values as FF numbers acquired over time. These operators offer more accurate depictions of evolving scenarios, facilitating well-informed decisions based on evolving knowledge.

### 1.3. Motivation

Dynamic aggregation operators are utilized to manage ambiguous data and uncertainties that undergo temporal variations. By facilitating the collection of data from multiple time periods, these operators enable a comprehensive comprehension of the issues at hand through the provision of an accurate depiction. This concept evaluates the information preference of a decision-maker across time periods by employing a time degree function. The introduction of the variable t in the dynamic system facilitates the monitoring of temporal variations in membership degrees. Therefore, changes within specified time periods can be analyzed. This functionality improves the accuracy of decisions, illuminates modifications, and assesses the dynamics of fuzzy sets. Dynamic weighted aggregation operators are important because they offer a flexible framework for combining the performance of data at different time periods in decision-making processes. Recently, the authors [49] conducted research on dynamic ordered weighted aggregation operators under complex IF knowledge. Alghazzawi et al. [50] conducted a study on dynamic aggregation operators within FF settings. Moreover, ordered weighted aggregation operators are not reliant on a preassigned weight for a specific attribute. These operators facilitate the rearrangement of input values, emphasizing their significance in the decision-making process. This enables decision-makers to articulate their choices without being constrained by exact weight values. These research obstacles emphasize the significance of establishing dynamic ordered weighted aggregation operators in the context of FF knowledge. Dynamic ordered weighted aggregation operators enhance decision-making accuracy and flexibility as compared to dynamic weighted aggregation operators.

Getting motivation from the preceding discourse, the present research introduces two novel dynamic ordered weighted aggregation operators within the framework of FF knowledge, namely, the Fermatean fuzzy dynamic ordered weighted averaging (FFDOWA) and the Fermatean fuzzy dynamic ordered weighted geometric (FFDOWG) operators, for the purpose of aggregating FF data in the framework of MADM problems. These novel operators offer more accurate depictions of developing situations, enabling educated judgments based on dynamic knowledge. They allow for the recording of changes over time and patterns of behavior within imprecise categories, beyond the capacity of other operators specifically Dynamic weighted aggregation operators.

The main objectives of the current study are delineated as:

- To introduce FF Dynamic ordered weighted aggregation operators, and to explore structural properties of these operators.
- To develop an algorithm for MADM problems by employing FF Dynamic ordered weighted aggregation operators.
- To address a particular MADM problem that focuses on determining the most effective big data analytics platform for YouTube data analysis using proposed techniques.
- To conduct a comparative analysis, that demonstrates the efficacy of suggested methodologies with existing strategies.

The following describes the main contributions made by this study:

- Two novel aggregation operators, namely the FFDOWA operator and the FFDOWG operator, are proposed. Moreover, many fundamental properties of these operators are investigated.
- A systematic methodology is devised to address MADM problems utilizing newly defined operators. This shows the practical implications of the suggested operators.
- The recommended approach is applied to the MADM problem to determine the most effective big data analytics platform for YouTube data analysis.
- A comprehensive comparative analysis is conducted to evaluate the efficacy of proposed methods in comparison to existing techniques.

The subsequent section of this manuscript is structured as follows: Section 2 furnishes requisite background definitions essential for comprehending the key findings outlined in this paper. Section 3 introduces dynamic aggregation operators designed for FFS and examines their inherent properties. Section 4 expounds upon a methodology to tackle the problem of MADM employing FF information through the utilization of Fermatean fuzzy dynamic ordered weighted aggregation operators. Section 5 of this article presents a case study on big data analytics tools, where newly developed operators are used to determine the most effective big data analytics platform for analyzing YouTube data. Moreover, this section also includes comparative study to showcase the efficacy and feasibility of these innovative methodologies in comparison to traditional strategies. Section 6 encapsulates a summary delineating the conclusions derived from the of this study.

## 2. Preliminaries

This section provides fundamental definitions that are essential for a thorough comprehension of the subject matter addressed in this article.

**Definition 1.** ([20]). Within the framework of a universe of discourse U, FFS ℱ over U is defined as $F=\{\langle x,\alpha_{F}(x),\beta_{F}(x)\rangle :\;x\in U\}$, where, $\alpha_{F}:\;U\to [0,1]$ represents membership function and $\beta_{F}:\;U\to [0,1]$, represents non-membership function satisfying the constraint $0\leq \alpha_{F}^{3}(x)+\beta_{F}^{3}(x)\leq 1,\forall x\in U$.

The values $\alpha_{F}(x)$ and $\beta_{F}(x)$, denotes the degree of membership and non-membership of x∈U. Furthermore, the indeterminacy degree of x∈U with regard to ℱ is represented as $\pi_{F}(x)=\sqrt[{3}]{1-\alpha_{F}^{3}(x)-\beta_{F}^{3}(x)}$.

In order to streamline the representation, the degrees of membership and non-membership of x∈U are represented as $x=(\alpha_{F},\beta_{F})$, which is referred to as the Fermatean fuzzy number (FFN), where, *α*_ℱ_, *β*_ℱ_∈[0,1] and such that $0\leq \alpha_{F}^{3}+\beta_{F}^{3}\leq 1$.

**Definition 2.** ([20]). For any FFN ℱ = (*α*_ℱ_, *β*_ℱ_), two crucial functions are delineated as follows:

- The score function *S*(ℱ), is defined by the expression as $S(F)=\alpha_{F}^{3}-\beta_{F}^{3}$, where *S*(ℱ)∈[−1,1].
- The accuracy function A(F), is formulated as $A(F)=\alpha_{F}^{3}+\beta_{F}^{3}$, where A(F)∈[0,1].

The FFNs ℱ_1_ and ℱ_2_ satisfy the subsequent criteria for comparison:

- If *S*(ℱ_1_)>*S*(ℱ_2_), then ℱ_1_>ℱ_2_
- If *S*(ℱ_1_)<*S*(ℱ_2_), then ℱ_1_<ℱ_2_
- If $S(F_{1})=S(F_{2})$, then $A(F_{1})>A(F_{2})\Rightarrow F_{1}>F_{2},\;A(F_{1})<A(F_{2})\Rightarrow F_{1}<F_{2}$ and $A(F_{1})=A(F_{2})\Rightarrow F_{1}∼F_{2}$

**Definition 3.** ([49]). Consider time variable *t*. A Fermatean fuzzy variable, denoted as ℱ_*t*_ = (*α*_*t*_, *β*_*t*_), where *α*_*t*_∈[0,1], *β*_*t*_∈[0,1], and $\alpha_{t}^{3}+\beta_{t}^{3}\leq 1$.

Note that $F_{t_{1}},F_{t_{2}},\ldots ,F_{t_{p}}$ denote FFNs accumulated during distinct time periods $t_{1},t_{2},\ldots ,t_{p}$ for a given FF variable ℱ_*t*_.

**Definition 4.** ([49]). Let’s consider two FFNs $F_{t_{1}}=(\alpha_{t_{1}},\beta_{t_{1}})$ and $F_{t_{2}}=(\alpha_{t_{2}},\beta_{t_{2}})$. The fundamental operational principles defining their interrelation are outlined as follows:

- $F_{t_{1}}\leq F_{t_{2}}$, if $\alpha_{t_{1}}\leq \alpha_{t_{2}}$ and $\beta_{t_{1}}\leq \beta_{t_{2}}$
-
$F_{t_{1}}^{c}=(\beta_{t_{1}},\alpha_{t_{1}})$

**Definition 5.** ([49]). Let ℱ_*t*_ = (*α*_*t*_, *β*_*t*_), $F_{t_{1}}=(\alpha_{t_{1}},\beta_{t_{1}})$ and $F_{t_{2}}=(\alpha_{t_{2}},\beta_{t_{2}})$ denote the FFNs and *ξ*_*t*_>0. We express the basic dynamic operations in the following way.:

-
$F_{t_{1}}⊕F_{t_{2}}=(\sqrt[{3}]{\alpha_{t_{1}}^{3}+\alpha_{t_{2}}^{3}-\alpha_{t_{1}}^{3}\alpha_{t_{2}}^{3}},\;\beta_{t_{1}}\beta_{t_{2}})$

-
$F_{t_{1}}⊗F_{t_{2}}=(\alpha_{t_{1}}\alpha_{t_{2}},\sqrt[{3}]{\beta_{t_{1}}^{3}+\beta_{t_{2}}^{3}-\beta_{t_{1}}^{3}\beta_{t_{2}}^{3}})$

-
$\xi_{t}F_{t}=(\sqrt[{3}]{1-{\left(1-\alpha_{t}^{3}\right)}^{\xi_{t}}},\beta_{t}^{\xi_{t}})$

-
$F_{t}^{\xi_{t}}=(\alpha_{t}^{\xi_{t}},\sqrt[{3}]{1-{\left(1-\beta_{t}^{3}\right)}^{\xi_{t}}})$

**Definition 6.** ([17]). Assume that *ψ* is a collection of FFNs ℱ_*j*_ = (*α*_*j*_, *β*_*j*_), where *j* = 1,2,…,*n*, and *γ* = (*γ*_1_, *γ*_2_,…,*γ*_*n*_)^*T*^ is the weight vector corresponding to these FFNs, such that *γ*_*j*_∈[0,1] and $\sum_{j=1}^{n}\gamma_{j}=1$. Then the Fermatean fuzzy weighted averaging (FFWA) operator is a function *FFWA*:*ψ*^*n*^→*ψ*, defined as:

$$FFWA(F_{1},F_{2},\ldots ,F_{n})=(\sum_{j=1}^{n}\gamma_{j}\alpha_{j},\sum_{j=1}^{n}\gamma_{j}\beta_{j}).$$

**Definition 7.** ([22]). Assume that *ψ* is a collection of FFNs ℱ_*j*_ = (*α*_*j*_, *β*_*j*_), where *j* = 1,2,…,*n*, and *γ* = (*γ*_1_, *γ*_2_,…,*γ*_*n*_)^*T*^ is the weight vector corresponding to these FFNs, such that *γ*_*j*_∈[0,1] and $\sum_{j=1}^{n}\gamma_{j}=1$. Then the Fermatean fuzzy weighted geometric operator (FFWG) operator is a function *FFWG*:*ψ*^*n*^→*ψ*, defined as:

$$FFWG(F_{1},F_{2},\ldots ,F_{n})=\left(\prod_{j=1}^{n}\alpha_{j}^{\gamma_{j}},\prod_{i=1}^{n}\beta_{j}^{\gamma_{j}}\right)$$

## 3. Dynamic ordered weighted aggregation operators for FFNs

This section explores the dynamic operations within Fermatean Fuzzy (FF) framework. In addition, we introduce dynamic ordered weighted aggregation operators for FFNs namely, FFDOWA and FFDOWG, and investigate their fundamental aspects.

### 3.1. Fundamental characteristics of Fermatean fuzzy dynamic ordered weighted averaging operator

In this section, we introduce FFDOWA operator and prove its fundamental properties.

**Definition 8.** Assume that *ψ* is a collection of FFNs $F_{t_{k}}=(\alpha_{t_{k}},\beta_{t_{k}})$, where k=1,2,…,p, at p different periods *t*_*k*_. Moreover, $\xi_{t}={\left[\xi_{t_{1}},\xi_{t_{2}},\ldots ,\xi_{t_{p}}\right]}^{T}$ is the associated weight vector of the time periods *t*_*k*_, such that $\xi_{t_{k}}\in [0,1]$ and $\sum_{k=1}^{p}\xi_{t_{k}}=1$. Then the Fermatean fuzzy dynamic ordered weighted averaging (FFDOWA) is a function $FFDOWA:\;\psi^{p}\to \psi$, defined as:

$$FFDOWA(F_{t_{1}}{,F}_{t_{2}},\ldots ,F_{t_{p}})={⊕}_{k=1}^{p}\xi_{t_{k}}F_{\sigma (t_{k})}$$

$$=\left(\sqrt[{3}]{1-\prod_{k=1}^{p}{\left(1-\alpha_{\sigma (t_{k})}^{3}\right)}^{\xi_{t_{k}}}},\prod_{k=1}^{p}\beta_{\sigma (t_{k})}^{\xi_{t_{k}}}\right)$$

Here, $\left(\sigma (t_{1}),\sigma (t_{2}),\ldots ,\sigma (t_{p})\right)$ denotes the permutation of $t_{k}=t_{1},t_{2},\ldots ,t_{p}$ such that $F_{\sigma (t_{k-1})}\geq F_{\sigma (t_{k})}$, for all *k*.

**Theorem 1.** Let k=1,2,…,p and $F_{t_{k}}=(\alpha_{t_{k}},\beta_{t_{k}})$ be a set of FFNs at p distinct time periods *t*_*k*_. Moreover, $\xi_{t}={\left[\xi_{t_{1}},\xi_{t_{2}},\ldots ,\xi_{t_{p}}\right]}^{T}$ is the weight vector corresponding to *t*_*k*_, such that $\xi_{t_{k}}\in [0,1]$ and $\sum_{k=1}^{p}\xi_{t_{k}}=1$. The aggregated outcome of these $F_{t_{k}}$ under the FFDOWA operator is a FFN. Mathematically,

$$FFDOWA(F_{t_{1}}{,F}_{t_{2}},\ldots ,F_{t_{p}})=\left(\sqrt[{3}]{1-\prod_{k=1}^{p}{\left(1-\alpha_{\sigma (t_{k})}^{3}\right)}^{\xi_{t_{k}}}},\prod_{k=1}^{p}\beta_{\sigma (t_{k})}^{\xi_{t_{k}}}\right)$$

Here, $\left(\sigma (t_{1}),\sigma (t_{2}),\ldots ,\sigma (t_{p})\right)$ denotes the permutation of $t_{k}=t_{1},t_{2},\ldots ,t_{p}$ such that $F_{\sigma (t_{k-1})}\geq F_{\sigma (t_{k})}$, for all *k*.

**Proof.** The proof of this theorem is accomplished by employing mathematical induction on p. Suppose that p=2, then

$$FFDOWA(F_{t_{1}},F_{t_{2}})=\xi_{t_{1}}.F_{\sigma (t_{1})}⊕\xi_{t_{2}}.F_{\sigma (t_{2})}$$

By applying Definition 8, the subsequent expressions can be derived:

$$\xi_{t_{1}}.F_{\sigma (t_{1})}=(\sqrt[{3}]{1-{\left(1-\alpha_{\sigma (t_{1})}^{3}\right)}^{\xi_{t_{1}}}},\beta_{\sigma (t_{1})}^{\xi_{t_{1}}})$$

$$\xi_{t_{2}}.F_{\sigma (t_{2})}=(\sqrt[{3}]{1-{\left(1-\alpha_{\sigma (t_{2})}^{3}\right)}^{\xi_{t_{2}}}},\beta_{\sigma (t_{2})}^{\xi_{t_{2}}})$$

Then,

$$\xi_{t_{1}}.F_{\sigma (t_{1})}⊕\xi_{t_{2}}.F_{\sigma (t_{2})}=(\sqrt[{3}]{1-{\left(1-\alpha_{\sigma (t_{1})}^{3}\right)}^{\xi_{t_{1}}}},\beta_{\sigma (t_{1})}^{\xi_{t_{1}}})⊕(\sqrt[{3}]{1-{\left(1-\alpha_{\sigma (t_{2})}^{3}\right)}^{\xi_{t_{2}}}},\beta_{\sigma (t_{2})}^{\xi_{t_{2}}})=(\sqrt[{3}]{1-{\left(1-\alpha_{\sigma (t_{1})}^{3}\right)}^{\xi_{t_{1}}}{\left(1-\alpha_{\sigma (t_{2})}^{3}\right)}^{\xi_{t_{2}}}},\beta_{\sigma (t_{1})}^{\xi_{t_{1}}}\beta_{\sigma (t_{2})}^{\xi_{t_{2}}})$$

Consequently,

$$FFDOWA(F_{t_{1}},F_{t_{2}})=(\sqrt[{3}]{1-\prod_{k=1}^{2}{\left(1-\alpha_{\sigma (t_{k})}^{3}\right)}^{\xi_{t_{k}}}},\prod_{k=1}^{2}\beta_{\sigma (t_{k})}^{\xi_{t_{k}}})$$

This means that the statement is true for p=2. Now we proceed by assuming that the assertion is correct for p=n>2, leading us to:

$$FFDOWA(F_{t_{1}},F_{t_{2}},\ldots ,F_{t_{n}})={⊕}_{k=1}^{n}\xi_{t_{k}}F_{\sigma (t_{k})}$$

$$=\left(\sqrt[{3}]{1-\prod_{k=1}^{n}{\left(1-\alpha_{\sigma (t_{k})}^{3}\right)}^{\xi_{t_{k}}}},\prod_{k=1}^{n}\beta_{\sigma (t_{k})}^{\xi_{t_{k}}}\right)$$

Now, for the scenario p=n+1, we may assess it as:

$$FFDOWA(F_{t_{1}},F_{t_{2}},\ldots ,F_{t_{n}},F_{t_{n+1}})={⊕}_{k=1}^{n}\xi_{t_{k}}F_{\sigma (t_{k})}⊕\xi_{t_{n+1}}F_{\sigma (t_{n+1})}=(\sqrt[{3}]{1-\prod_{k=1}^{n}{\left(1-\alpha_{\sigma (t_{k})}^{3}\right)}^{\xi_{t_{k}}}},\prod_{k=1}^{n}\beta_{\sigma (t_{k})}^{\xi_{t_{k}}})⊕(\sqrt[{3}]{1-{\left(1-\alpha_{\sigma (t_{n+1})}^{3}\right)}^{\xi_{t_{n+1}}}},\beta_{\sigma (t_{n+1})}^{\xi_{t_{n+1}}})$$

This implies that

$$FFDOWA(F_{t_{1}},F_{t_{2}},\ldots ,F_{t_{n+1}})=\left(\sqrt[{3}]{1-\prod_{k=1}^{n+1}{\left(1-\alpha_{\sigma (t_{k})}^{3}\right)}^{\xi_{t_{k}}}},\prod_{k=1}^{n+1}\beta_{\sigma (t_{k})}^{\xi_{t_{k}}}\right)$$

This proves the statement for p=n+1. Thus, the statement is valid for all $p\in Z^{+}$.

The example that follows explains Theorem 1.

**Example 1.** Let $F_{t_{1}}=(0.70,0.60),\;F_{t_{2}}=(0.80,0.40),\;F_{t_{3}}=(0.90,0.50)$ and $F_{t_{4}}=(0.80,0.70)$ be four FFNs where, $\xi_{t}={\left(0.10,0.20,0.30,0.40\right)}^{T}$ be the associated weighted vector of *t*_1_, *t*_2_, *t*_3_, and *t*_4_. First, let us compute the scores of $F_{t_{k}}$, using Definition 2 in the following manners:

$$S(F_{t_{1}})=0.127,\;S(F_{t_{2}})=0.448$$

$$S(F_{t_{3}})=0.604,\;S(F_{t_{4}})=0.169$$

Then the permuted values of FFNs are arranged as follows:

$$F_{\sigma (t_{1})}=(0.90,0.50),\;F_{\sigma (t_{2})}=(0.80,0.40),\;F_{\sigma (t_{3})}=(0.80,0.70),\;F_{\sigma (t_{4})}=(0.70,0.60)$$

Now, we aggregate the above obtained values within the framework of Definition 8 as follows:

$$FFDOWA(F_{t_{1}},F_{t_{2}},F_{t_{3}},F_{t_{4}})=\left(\sqrt[{3}]{1-\prod_{k=1}^{4}{\left(1-\alpha_{\sigma (t_{k})}^{3}\right)}^{\xi_{t_{k}}}},\prod_{k=1}^{4}\beta_{\sigma (t_{k})}^{\xi_{t_{k}}}\right)$$

$$=\left(\begin{array}{c} \sqrt[{3}]{1-{\left(1-{\left(0.9\right)}^{3}\right)}^{0.1}{\left(1-{\left(0.8\right)}^{3}\right)}^{0.2}{\left(1-{\left(0.8\right)}^{3}\right)}^{0.3}{\left(1-{\left(0.7\right)}^{3}\right)}^{0.4}},\\ {\left(0.5\right)}^{0.1}{\left(0.4\right)}^{0.2}{\left(0.7\right)}^{0.3}{\left(0.6\right)}^{0.4} \end{array}\right)$$

Consequently,

$$FFDOWA(F_{t_{1}},F_{t_{2}},F_{t_{3}},F_{t_{4}})=(0.783,0.568)$$

**Theorem 2.** (Idempotency) Let k=1,2,…,p and $F_{t_{k}}=(\alpha_{t_{k}},\beta_{t_{k}})$ be a set of FFNs at p distinct time periods *t*_*k*_. Moreover, $\xi_{t}={\left[\xi_{t_{1}},\xi_{t_{2}},\ldots ,\xi_{t_{p}}\right]}^{T}$ is the weight vector corresponding to *t*_*k*_ satisfying that $\xi_{t_{k}}\in [0,1]$ and $\sum_{k=1}^{p}\xi_{t_{k}}=1$. If, $F_{\sigma (t_{k})}=F_{\sigma (t_{j})}=\left(\alpha_{\sigma (t_{j})},\beta_{\sigma (t_{j})}\right)$ are identical for all *k* = 1,2,…,*p*, and for some j∈{1,2,…,p} then,

$$FFDOWA(F_{t_{1}}{,F}_{t_{2}},\ldots ,F_{t_{p}})=F_{\sigma (t_{j})}$$

Here, $\left(\sigma (t_{1}),\sigma (t_{2}),\ldots ,\sigma (t_{p})\right)$ denotes the permutation of $t_{k}=t_{1},t_{2},\ldots ,t_{p}$ such that $F_{\sigma (t_{k-1})}\geq F_{\sigma (t_{k})}$, for all *k*.

**Proof.** In view of the given fact, we have $\alpha_{\sigma (t_{k})}=\alpha_{\sigma (t_{j})}$ and $\beta_{\sigma (t_{k})}=\beta_{\sigma (t_{j})}$, then the application of above relations in Definition 8 yields the following outcomes

$$FFDOWA(F_{t_{1}}{,F}_{t_{2}},\ldots ,F_{t_{p}})=\left(\sqrt[{3}]{1-{\left(1-\alpha_{\sigma (t_{j})}^{3}\right)}^{\sum_{k=1}^{p}\xi_{t_{k}}}},\beta_{\sigma (t_{j})}^{\sum_{k=1}^{p}\xi_{t_{k}}}\right)$$

$$=(\sqrt[{3}]{1-(1-\alpha_{\sigma (t_{j})}^{3})},\beta_{\sigma (t_{j})})=(\sqrt[{3}]{\alpha_{\sigma (t_{j})}^{3}},\beta_{\sigma (t_{j})})=(\alpha_{\sigma (t_{j})},\beta_{\sigma (t_{j})})$$

Consequently,

$$FFDOWA(F_{t_{1}}{,F}_{t_{2}},\ldots ,F_{t_{p}})=F_{\sigma (t_{j})}$$

**Theorem 3.** (Boundedness) Let $F_{t}^{-}=\left(\underset{t_{k}}{\min}\left\{\alpha_{\sigma (t_{k})}\right\},\underset{t_{k}}{\max}\{\beta_{\sigma (t_{k})}\}\right)$ and $F_{t}^{+}=\left(\underset{t_{k}}{\max}\left\{\alpha_{\sigma (t_{k})}\right\},\underset{t_{k}}{\min}\left\{\beta_{\sigma (t_{k})}\right\}\right)$ are respectively the lower and upper bounds of the FFNs $F_{t_{k}}=(\alpha_{t_{k}},\beta_{t_{k}})$, where *k* takes on values from 1 to p and $\xi_{t}={\left[\xi_{t_{1}},\xi_{t_{2}},\ldots ,\xi_{t_{p}}\right]}^{T}$ be the weight vector corresponding to *t*_*k*_ satisfying $\xi_{t_{k}}\in [0,1]$ and $\sum_{k=1}^{p}\xi_{t_{k}}=1$. Moreover, $\left(\sigma (t_{1}),\sigma (t_{2}),\ldots ,\sigma (t_{p})\right)$ denotes the permutation of $t_{k}=t_{1},t_{2},\ldots ,t_{p}$ such that $F_{\sigma (t_{k-1})}\geq F_{\sigma (t_{k})}$, for all *k*. Then,

$${F_{t}}^{-}\leq FFDOWA(F_{t_{1}}{,F}_{t_{2}},\ldots ,F_{t_{p}})\leq {F_{t}}^{+}$$

**Proof.** Consider the result of applying the FFDOWA operator to the collection of FFNs, as follows:

$$FFDOWA(F_{t_{1}}{,F}_{t_{2}},\ldots ,F_{t_{p}})=(\alpha_{t},\beta_{t})$$

In view of the given condition, we have

$$\underset{t_{k}}{\min}\left\{\alpha_{\sigma (t_{k})}\right\}\leq \alpha_{\sigma (t_{k})}\leq \underset{t_{k}}{\max}\left\{\alpha_{\sigma (t_{k})}\right\}$$

$$\Rightarrow \underset{t_{k}}{\min}\left\{\alpha_{\sigma (t_{k})}^{3}\right\}\leq \alpha_{\sigma (t_{k})}^{3}\leq \underset{t_{k}}{\max}\left\{\alpha_{\sigma (t_{k})}^{3}\right\}$$

$$\Rightarrow 1-\underset{t_{k}}{\max}\left\{\alpha_{\sigma (t_{k})}^{3}\right\}\leq 1-\alpha_{\sigma (t_{k})}^{3}\leq 1-\underset{t_{k}}{\min}\left\{\alpha_{\sigma (t_{k})}^{3}\right\}$$

$$\Rightarrow \prod_{k=1}^{p}{\left(1-\underset{t_{k}}{\max}\left\{\alpha_{\sigma (t_{k})}^{3}\right\}\right)}^{\xi_{t_{k}}}\leq \prod_{k=1}^{p}{\left(1-\alpha_{\sigma (t_{k})}^{3}\right)}^{\xi_{t_{k}}}\leq \prod_{k=1}^{p}{\left(1-\underset{t_{k}}{\min}\left\{\alpha_{\sigma (t_{k})}^{3}\right\}\right)}^{\xi_{t_{k}}}$$

$$\Rightarrow {\left(1-\underset{t_{k}}{\max}\left\{\alpha_{\sigma (t_{k})}^{3}\right\}\right)}^{\sum_{k=1}^{p}\xi_{t_{k}}}\leq \prod_{k=1}^{p}{\left(1-\alpha_{\sigma (t_{k})}^{3}\right)}^{\xi_{t_{k}}}\leq {\left(1-\underset{t_{k}}{\min}\left\{\alpha_{\sigma (t_{k})}^{3}\right\}\right)}^{\sum_{k=1}^{p}\xi_{t_{k}}}$$

$$\Rightarrow \left(1-\underset{t_{k}}{\max}\left\{\alpha_{\sigma (t_{k})}^{3}\right\}\right)\leq \prod_{k=1}^{p}{\left(1-\alpha_{\sigma (t_{k})}^{3}\right)}^{\xi_{t_{k}}}\leq \left(1-\underset{t_{k}}{\min}\left\{\alpha_{\sigma (t_{k})}^{3}\right\}\right)$$

$$\Rightarrow \underset{t_{k}}{\min}\left\{\alpha_{\sigma (t_{k})}^{3}\right\}\leq 1-\prod_{k=1}^{p}{\left(1-\alpha_{\sigma (t_{k})}^{3}\right)}^{\xi_{t_{k}}}\leq \underset{t_{k}}{\max}\left\{\alpha_{\sigma (t_{k})}^{3}\right\}$$

$$\Rightarrow \sqrt[{3}]{\underset{t_{k}}{\min}\left\{\alpha_{\sigma (t_{k})}^{3}\right\}}\leq \sqrt[{3}]{1-\prod_{k=1}^{p}{\left(1-\alpha_{\sigma (t_{k})}^{3}\right)}^{\xi_{t_{k}}}}\leq \sqrt[{3}]{\underset{t_{k}}{\max}\left\{\alpha_{\sigma (t_{k})}^{3}\right\}}$$

$$\Rightarrow \underset{t_{k}}{\min}\left\{\alpha_{\sigma (t_{k})}\right\}\leq \alpha_{t}\leq \underset{t_{k}}{\max}\left\{\alpha_{\sigma (t_{k})}\right\}$$ (1)

Moreover,

$$\underset{t_{k}}{\min}\left\{\beta_{\sigma (t_{k})}\right\}\leq \beta_{\sigma (t_{k})}\leq \underset{t_{k}}{\max}\left\{\beta_{\sigma (t_{k})}\right\}$$

$$\Rightarrow \prod_{k=1}^{p}{\left(\underset{t_{k}}{\min}\left\{\beta_{\sigma (t_{k})}\right\}\right)}^{\xi_{t_{k}}}\leq \prod_{k=1}^{p}{\left(\beta_{\sigma (t_{k})}\right)}^{\xi_{t_{k}}}\leq \prod_{k=1}^{p}{\left(\underset{t_{k}}{\max}\left\{\beta_{\sigma (t_{k})}\right\}\right)}^{\xi_{t_{k}}}$$

$$\Rightarrow {\left(\underset{t_{k}}{\min}\left\{\beta_{\sigma (t_{k})}\right\}\right)}^{\sum_{k=1}^{p}\xi_{t_{k}}}\leq \prod_{k=1}^{p}{\left(\beta_{\sigma (t_{k})}\right)}^{\xi_{t_{k}}}\leq {\left(\underset{t_{k}}{\max}\left\{\beta_{\sigma (t_{k})}\right\}\right)}^{\sum_{k=1}^{p}\xi_{t_{k}}}$$

$$\Rightarrow \underset{t_{k}}{\min}\left\{\beta_{\sigma (t_{k})}\right\}\leq \beta_{t}\leq \underset{t_{k}}{\max}\left\{\beta_{\sigma (t_{k})}\right\}$$

Comparing inequalities 1 and 2 yields the following result:

$${F_{t}}^{-}\leq FFDOWA(F_{t_{1}}{,F}_{t_{2}},\ldots ,F_{t_{p}})\leq {F_{t}}^{+}.$$ (2)

**Theorem 4.** (Monotonicity) Let $F_{t_{k}}=(\alpha_{t_{k}},\beta_{t_{k}})$ and $F_{t_{k}}^{\prime }=(\alpha_{t_{k}}^{\prime },\beta_{t_{k}}^{\prime })$, where k=1,2,…,p, be the sets of FFNs. Suppose that $\xi_{t}={\left[\xi_{t_{1}},\xi_{t_{2}},\ldots ,\xi_{t_{p}}\right]}^{T}$ is the weight vector corresponding to *t*_*k*_ satisfying $\xi_{t_{k}}\in [0,1]$ and $\sum_{k=1}^{p}\xi_{t_{k}}=1$. In addition, $\left(\sigma (t_{1}),\sigma (t_{2}),\ldots ,\sigma (t_{p})\right)$ denotes the permutation of $t_{k}=t_{1},t_{2},\ldots ,t_{p}$ such that $F_{\sigma (t_{k-1})}\geq F_{\sigma (t_{k})}$, for all *k*. If $\alpha_{\sigma (t_{k})}\leq \alpha_{\sigma (t_{k})}^{\prime }$ and $\beta_{\sigma (t_{k})}\geq \beta_{\sigma (t_{k})}^{\prime }$, then we can establish that:

$$FFDOWA(F_{t_{1}}{,F}_{t_{2}},\ldots ,F_{t_{p}})\leq FFDOWA(F_{t_{1}}^{\prime },F_{t_{2}}^{\prime },\ldots ,F_{t_{p}}^{\prime })$$

**Proof.** Let us apply FFDOWA operator on the given collections of FFNs as follows:

$$FFDOWA(F_{t_{1}}{,F}_{t_{2}},\ldots ,F_{t_{p}})=(\alpha_{t},\beta_{t})andFFDOWA(F_{t_{1}}^{\prime },F_{t_{2}}^{\prime },\ldots ,F_{t_{p}}^{\prime })=(\alpha_{t}^{\prime },\beta_{t}^{\prime })$$

Since $\alpha_{\sigma (t_{k})}\leq \alpha_{\sigma (t_{k})}^{\prime }$, which implies that $\alpha_{\sigma (t_{k})}^{3}\leq \alpha_{\sigma (t_{k})}^{\prime^{3}}$, we can deduce that

$$1-\alpha_{\sigma (t_{k})}^{3}\geq 1-\alpha_{\sigma (t_{k})}^{\prime^{3}}$$

$$\Rightarrow \prod_{k=1}^{p}{\left(1-\alpha_{\sigma (t_{k})}^{3}\right)}^{\xi_{t_{k}}}\geq \prod_{k=1}^{p}{\left(1-\alpha_{\sigma (t_{k})}^{\prime^{3}}\right)}^{\xi_{t_{k}}}$$

$$\Rightarrow 1-\prod_{k=1}^{p}{\left(1-\alpha_{\sigma (t_{k})}^{3}\right)}^{\xi_{t_{k}}}\leq 1-\prod_{k=1}^{p}{\left(1-\alpha_{\sigma (t_{k})}^{\prime^{3}}\right)}^{\xi_{t_{k}}}$$

$$\Rightarrow \sqrt[{3}]{1-\prod_{k=1}^{p}{\left(1-\alpha_{\sigma (t_{k})}^{3}\right)}^{\xi_{t_{k}}}}\leq \sqrt[{3}]{1-\prod_{k=1}^{p}{\left(1-\alpha_{\sigma (t_{k})}^{\prime^{3}}\right)}^{\xi_{t_{k}}}}$$

Hence, we can conclude that

$$\alpha_{t}\leq \alpha_{t}^{\prime }$$ (3)

Similarly, by considering $\beta_{\sigma (t_{k})}\geq \beta_{\sigma (t_{k})}^{\prime }$, we derive:

$$\prod_{k=1}^{p}\beta_{\sigma (t_{k})}^{\xi_{t_{k}}}\geq \prod_{k=1}^{p}\beta_{\sigma (t_{k})}^{\prime^{\xi_{t_{k}}}}$$

Which implies,

$$\beta_{t}\geq \beta_{t}^{\prime }$$ (4)

Thus, from the comparison of 3 and 4 and the application of Definition 4, we can deduce that

$$FFDOWA(F_{t_{1}}{,F}_{t_{2}},\ldots ,F_{t_{p}})\leq FFDOWA(F_{t_{1}}^{\prime },F_{t_{2}}^{\prime },\ldots ,F_{t_{p}}^{\prime })$$

Thus, the monotonicity property is established.

### 3.2. Fundamental characteristics of Fermatean fuzzy dynamic ordered weighted geometric operator

This section constitutes an introduction to the FFDOWG operator for FFNs and an examination of its structural attributes.

**Definition 9.** Assume that *ψ* is a collection of FFNs $F_{t_{k}}=(\alpha_{t_{k}},\beta_{t_{k}})$, where k=1,2,…,p, at p different periods *t*_*k*_. Moreover, $\xi_{t}={\left[\xi_{t_{1}},\xi_{t_{2}},\ldots ,\xi_{t_{p}}\right]}^{T}$ is the associated weight vector of the time periods *t*_*k*_, such that $\xi_{t_{k}}\in [0,1]$ and $\sum_{k=1}^{p}\xi_{t_{k}}=1$. Then the Fermatean fuzzy dynamic ordered weighted geometric (FFDOWG) is a function $FFDOWG:\psi^{p}\to \psi$, defined as:

$$FFDOWG(F_{t_{1}}{,F}_{t_{2}},\ldots ,F_{t_{p}})={⊗}_{k=1}^{p}F_{\sigma (t_{k})}^{\xi_{t_{k}}}$$

$$=\left(\prod_{k=1}^{p}\alpha_{\sigma (t_{k})}^{\xi_{t_{k}}},\sqrt[{3}]{1-\prod_{k=1}^{p}{\left(1-\beta_{\sigma (t_{k})}^{3}\right)}^{\xi_{t_{k}}}}\right)$$

Here, $\left(\sigma (t_{1}),\sigma (t_{2}),\ldots ,\sigma (t_{p})\right)$ denotes the permutation of $t_{k}=t_{1},t_{2},\ldots ,t_{p}$ such that $F_{\sigma (t_{k-1})}\geq F_{\sigma (t_{k})}$, for all *k*.

**Theorem 5.** Let $F_{t_{k}}=(\alpha_{t_{k}},\beta_{t_{k}})$ be set of FFNs, where k=1,2,…,p, existing at p distinct time periods *t*_*k*_ and $\xi_{t}={\left[\xi_{t_{1}},\xi_{t_{2}},\ldots ,\xi_{t_{p}}\right]}^{T}$ be the weight vector corresponding to *t*_*k*_ satisfying $\xi_{t_{k}}\in [0,1]$ and $\sum_{k=1}^{p}\xi_{t_{k}}=1$. The aggregated outcome of these $F_{t_{k}}$ using FFDOWG operator remains an FFN. It can be expressed as:

$$FFDOWG(F_{t_{1}}{,F}_{t_{2}},\ldots ,F_{t_{p}})=\left(\prod_{k=1}^{p}\alpha_{\sigma (t_{k})}^{\xi_{t_{k}}},\sqrt[{3}]{1-\prod_{k=1}^{p}{\left(1-\beta_{\sigma (t_{k})}^{3}\right)}^{\xi_{t_{k}}}}\right)$$

Here, $\left(\sigma (t_{1}),\sigma (t_{2}),\ldots ,\sigma (t_{p})\right)$ denotes the permutation of $t_{k}=t_{1},t_{2},\ldots ,t_{p}$ such that $F_{\sigma (t_{k-1})}\geq F_{\sigma (t_{k})}$, for all *k*.

**Proof.** The proof of this theorem is accomplished by employing mathematical induction on p. Suppose that p=2, then

$$FFDOWG(F_{t_{1}}{,F}_{t_{2}})=F_{\sigma (t_{1})}^{\xi_{t_{1}}}⊗F_{\sigma (t_{2})}^{\xi_{t_{2}}}$$

Breaking down the components $F_{\sigma (t_{1})}^{\xi_{t_{1}}}$ and $F_{\sigma (t_{2})}^{\xi_{t_{2}}}$, by applying Definition 9 the subsequent expressions can be derived:

$$F_{\sigma (t_{1})}^{\xi_{t_{1}}}=\left(\alpha_{\sigma (t_{1})}^{\xi_{t_{1}}},\sqrt[{3}]{1-{\left(1-\beta_{\sigma (t_{1})}^{3}\right)}^{\xi_{t_{1}}}}\right)$$

$$F_{\sigma (t_{2})}^{\xi_{t_{2}}}=\left(\alpha_{\sigma (t_{2})}^{\xi_{t_{2}}},\sqrt[{3}]{1-{\left(1-\beta_{\sigma (t_{2})}^{3}\right)}^{\xi_{t_{2}}}}\right)$$

Then,

$$F_{\sigma (t_{1})}^{\xi_{t_{1}}}⊗F_{\sigma (t_{2})}^{\xi_{t_{2}}}=\left(\alpha_{\sigma (t_{1})}^{\xi_{t_{1}}},\sqrt[{3}]{1-{\left(1-\beta_{\sigma (t_{1})}^{3}\right)}^{\xi_{t_{1}}}}\right)⊗\left(\alpha_{\sigma (t_{2})}^{\xi_{t_{2}}},\sqrt[{3}]{1-{\left(1-\beta_{\sigma (t_{2})}^{3}\right)}^{\xi_{t_{2}}}}\right)=\left(\alpha_{\sigma (t_{1})}^{\xi_{t_{1}}}\alpha_{\sigma (t_{2})}^{\xi_{t_{2}}},\sqrt[{3}]{1-{{{(1-\beta }_{\sigma (t_{1})}}^{3})}^{\xi_{t_{1}}}{{{(1-\beta }_{\sigma (t_{2})}}^{3})}^{\xi_{t_{2}}}}\right)$$

Consequently,

$$FFDOWG(F_{t_{1}},F_{t_{2}})=\left(\prod_{k=1}^{2}\alpha_{\sigma (t_{k})}^{\xi_{t_{k}}},\sqrt[{3}]{1-\prod_{k=1}^{2}{\left(1-\beta_{\sigma (t_{k})}^{3}\right)}^{\xi_{t_{k}}}}\right)$$

This means that the statement is true for p=2. Now we proceed by assuming that the assertion is correct for p=n>2, leading us to:

$$FFDOWG(F_{t_{1}}{,F}_{t_{2}},\ldots ,F_{n})={⊗}_{k=1}^{n}F_{\sigma (t_{k})}^{\xi_{t_{k}}}$$

$$=\left(\prod_{k=1}^{n}\alpha_{\sigma (t_{k})}^{\xi_{t_{k}}},\sqrt[{3}]{1-\prod_{k=1}^{n}{\left(1-\beta_{\sigma (t_{k})}^{3}\right)}^{\xi_{t_{k}}}}\right)$$

Now, for the scenario p=n+1, we may assess it as:

$$FFDOWG(F_{t_{1}}{,F}_{t_{2}},\ldots ,F_{t_{n}},F_{t_{n+1}})={⊗}_{k=1}^{n}F_{\sigma (t_{k})}^{\xi_{t_{k}}}⊗F_{t_{n+1}}^{\xi_{t_{n+1}}}=\left(\prod_{k=1}^{n}\alpha_{\sigma (t_{k})}^{\xi_{t_{k}}},\sqrt[{3}]{1-\prod_{k=1}^{n}{\left(1-\beta_{\sigma (t_{k})}^{3}\right)}^{\xi_{t_{k}}}}\right)⊗\left(\alpha_{\sigma (t_{n+1})}^{\xi_{t_{n+1}}},\sqrt[{3}]{1-{\left(1-\beta_{\sigma (t_{n+1})}^{3}\right)}^{\xi_{t_{n+1}}}}\right)$$

This mean that

$$FFDOWG(F_{t_{1}}{,F}_{t_{2}},\ldots ,F_{t_{n+1}})=\left(\prod_{k=1}^{n+1}\alpha_{\sigma (t_{k})}^{\xi_{t_{k}}},\sqrt[{3}]{1-\prod_{k=1}^{n+1}{\left(1-\beta_{\sigma (t_{k})}^{3}\right)}^{\xi_{t_{k}}}}\right)$$

This proves the statement for p=n+1. Thus, the assertion is true for all $p\in Z^{+}$.

**Example 2.** Let $F_{t_{1}}=(0.80,0.70),\;F_{t_{2}}=(0.90,0.40),\;F_{t_{3}}=(0.70,0.50)$ and $F_{t_{4}}=(0.80,0.60)$ be four FFNs where, $\xi_{t}={\left(0.05,0.15,0.35,0.45\right)}^{T}$ be the weighted vector of time periods *t*_*k*_, where *k* = 1,2,3,4. First, we calculate the scores of $F_{t_{k}}$ using Definition 2, in the following way:

$$S(F_{t_{1}})=0.169,\;S(F_{t_{2}})=0.665$$

$$S(F_{t_{3}})=0.218,\;S(F_{t_{4}})=0.296$$

Then the permuted values of FFNs are arranged as follows:

$$F_{\sigma (t_{1})}=(0.90,0.40),\;F_{\sigma (t_{2})}=(0.80,0.60),\;F_{\sigma (t_{3})}=(0.70,0.50),\;F_{\sigma (t_{4})}=(0.80,0.70)$$

Thus, we aggregate the above obtained values in the framework of Definition 9 as follows:

$$FFDOWG(F_{t_{1}}{,F}_{t_{2}},F_{t_{3}},F_{t_{4}})=\left(\prod_{k=1}^{4}\alpha_{\sigma (t_{k})}^{\xi_{t_{k}}},\sqrt[{3}]{1-\prod_{k=1}^{4}{\left(1-\beta_{\sigma (t_{k})}^{3}\right)}^{\xi_{t_{k}}}}\right)$$

$$=\left(\begin{array}{c} {\left(0.9\right)}^{0.05}{\left(0.8\right)}^{0.15}{\left(0.7\right)}^{0.35}{\left(0.8\right)}^{0.45},\\ \sqrt[{3}]{1-{\left(1-{\left(0.4\right)}^{3}\right)}^{0.05}{\left(1-{\left(0.6\right)}^{3}\right)}^{0.15}{\left(1-{\left(0.5\right)}^{3}\right)}^{0.35}{\left(1-{\left(0.7\right)}^{3}\right)}^{0.45}} \end{array}\right)$$

Consequently,

$$FFDOWG(F_{t_{1}}{,F}_{t_{2}},F_{t_{3}},F_{t_{4}})=(0.767,0.622)$$

**Theorem 6.** (Idempotency) Consider p number of FFNs represented as $F_{t_{k}}=(\alpha_{t_{k}},\beta_{t_{k}})$, existing at p distinct time periods *t*_*k*_, where k=1,2,…,p and $\xi_{t}={\left[\xi_{t_{1}},\xi_{t_{2}},\ldots ,\xi_{t_{p}}\right]}^{T}$ is the weight vector corresponding to *t*_*k*_ satisfying $\xi_{t_{k}}\in [0,1]$ and $\sum_{k=1}^{p}\xi_{t_{k}}=1$. If, $F_{\sigma (t_{k})}=F_{\sigma (t_{j})}=(\alpha_{\sigma (t_{j})},\beta_{\sigma (t_{j})})$ are mathematically identical for all *k* = 1,2,…,*p*, and for some j∈{1,2,…,p} then,

$$FFDOWG(F_{t_{1}}{,F}_{t_{2}},\ldots ,F_{t_{p}})=F_{\sigma (t_{j})}$$

Here, $\left(\sigma (t_{1}),\sigma (t_{2}),\ldots ,\sigma (t_{p})\right)$ denotes the permutation of $t_{k}=t_{1},t_{2},\ldots ,t_{p}$ such that $F_{\sigma (t_{k-1})}\geq F_{\sigma (t_{k})}$, for all *k*.

**Proof.** In view of given fact, we have $\alpha_{\sigma (t_{k})}=\alpha_{\sigma (t_{j})}$ and $\beta_{\sigma (t_{k})}=\beta_{\sigma (t_{j})}$, then the application of above relations in Definition 9 yields the following outcomes:

$$FFDOWG(F_{t_{1}}{,F}_{t_{2}},\ldots ,F_{t_{p}})=\left(\alpha_{\sigma (t_{j})}^{\sum_{k=1}^{p}\xi_{t_{k}}},\sqrt[{3}]{1-{\left(1-\beta_{\sigma (t_{j})}^{3}\right)}^{\sum_{k=1}^{p}\xi_{t_{k}}}}\right)$$

$$=\left(\alpha_{\sigma (t_{j})},\sqrt[{3}]{1-(1-\beta_{\sigma (t_{j})}^{3})}\right)=\left(\alpha_{\sigma (t_{j})},\sqrt[{3}]{\beta_{\sigma (t_{j})}^{3}}\right)=\left(\alpha_{\sigma (t_{j})},\beta_{\sigma (t_{j})}\right)$$

Consequently,

$$FFDOWG(F_{t_{1}}{,F}_{t_{2}},\ldots ,F_{t_{p}})=F_{\sigma (t_{j})}$$

**Theorem 7.** (Boundedness) Let $F_{t_{k}}^{-}=\left(\underset{t_{k}}{\min}\left\{\alpha_{\sigma (t_{k})}\right\},\underset{t_{k}}{\max}\left\{\beta_{\sigma (t_{k})}\right\}\right)$ and $F_{t_{k}}^{+}=\left(\underset{t_{k}}{\max}\left\{\alpha_{\sigma (t_{k})}\right\},\underset{t_{k}}{\min}\left\{\beta_{\sigma (t_{k})}\right\}\right)$ are respectively lower and upper bounds of the FFNs $F_{t_{k}}=(\alpha_{t_{k}},\beta_{t_{k}})$, where *k* takes on values from 1 to p and $\xi_{t}={\left[\xi_{t_{1}},\xi_{t_{2}},\ldots ,\xi_{t_{p}}\right]}^{T}$ be the weight vector corresponding to *t*_*k*_ satisfying $\xi_{t_{k}}\in [0,1]$ and $\sum_{k=1}^{p}\xi_{t_{k}}=1$. Moreover, $\left(\sigma (t_{1}),\sigma (t_{2}),\ldots ,\sigma (t_{p})\right)$ denotes the permutation of $t_{k}=t_{1},t_{2},\ldots ,t_{p}$ such that $F_{\sigma (t_{k-1})}\geq F_{\sigma (t_{k})}$, for all *k*. Then

$${F_{t}}^{-}\leq FFDOWG(F_{t_{1}}{,F}_{t_{2}},\ldots ,F_{t_{p}})\leq {F_{t}}^{+}$$

**Proof.** The outcome of applying the FFDOWG operator to the given collection of FFNs can be described as follows:

$$FFDOWG(F_{t_{1}}{,F}_{t_{2}},\ldots ,F_{t_{p}})=(\alpha_{t},\beta_{t}).$$

In view of the given condition, we have

$$\underset{t_{k}}{\min}\left\{\alpha_{\sigma (t_{k})}\right\}\leq \alpha_{\sigma (t_{k})}\leq \underset{t_{k}}{\max}\left\{\alpha_{\sigma (t_{k})}\right\}$$

$$\Rightarrow \prod_{k=1}^{p}{\left(\underset{t_{k}}{\min}\left\{\alpha_{\sigma (t_{k})}\right\}\right)}^{\xi_{t_{k}}}\leq \prod_{k=1}^{p}{\left(\alpha_{\sigma (t_{k})}\right)}^{\xi_{t_{k}}}\leq \prod_{k=1}^{p}{\left(\underset{t_{k}}{\max}\left\{\alpha_{\sigma (t_{k})}\right\}\right)}^{\xi_{t_{k}}}$$

$$\Rightarrow {\left(\underset{t_{k}}{\min}\left\{\alpha_{\sigma (t_{k})}\right\}\right)}^{\sum_{k=1}^{p}\xi_{t_{k}}}\leq \prod_{k=1}^{p}{\left(\alpha_{\sigma (t_{k})}\right)}^{\xi_{t_{k}}}\leq {\left(\underset{t_{k}}{\max}\left\{\alpha_{\sigma (t_{k})}\right\}\right)}^{\sum_{k=1}^{p}\xi_{t_{k}}}$$

$$\Rightarrow \underset{t_{k}}{\min}\left\{\alpha_{\sigma (t_{k})}\right\}\leq \alpha_{t}\leq \underset{t_{k}}{\max}\left\{\alpha_{\sigma (t_{k})}\right\}$$ (5)

Moreover,

$$\underset{t_{k}}{\min}\left\{\beta_{\sigma (t_{k})}\right\}\leq \beta_{\sigma (t_{k})}\leq \underset{t_{k}}{\max}\left\{\beta_{\sigma (t_{k})}\right\}$$

$$\Rightarrow \underset{t_{k}}{\min}\left\{\beta_{\sigma (t_{k})}^{3}\right\}\leq \beta_{\sigma (t_{k})}^{3}\leq \underset{t_{k}}{\max}\left\{\beta_{\sigma (t_{k})}^{3}\right\}$$

$$\Rightarrow 1-\underset{t_{k}}{\max}\left\{\beta_{\sigma (t_{k})}^{3}\right\}\leq 1-\beta_{\sigma (t_{k})}^{3}\leq 1-\underset{t_{k}}{\min}\left\{\beta_{\sigma (t_{k})}^{3}\right\}$$

$$\Rightarrow \prod_{k=1}^{p}{\left(1-\underset{t_{k}}{\max}\left\{\beta_{\sigma (t_{k})}^{3}\right\}\right)}^{\xi_{t_{k}}}\leq \prod_{k=1}^{p}{\left(1-\beta_{\sigma (t_{k})}^{3}\right)}^{\xi_{t_{k}}}\leq \prod_{k=1}^{p}{\left(1-\underset{t_{k}}{\min}\left\{\beta_{\sigma (t_{k})}^{3}\right\}\right)}^{\xi_{t_{k}}}$$

$$\Rightarrow {\left(1-\underset{t_{k}}{\max}\left\{\beta_{\sigma (t_{k})}^{3}\right\}\right)}^{\sum_{k=1}^{p}\xi_{t_{k}}}\leq \prod_{k=1}^{p}{\left(1-\beta_{\sigma (t_{k})}^{3}\right)}^{\xi_{t_{k}}}\leq {\left(1-\underset{t_{k}}{\min}\left\{\beta_{\sigma (t_{k})}^{3}\right\}\right)}^{\sum_{k=1}^{p}\xi_{t_{k}}}$$

$$\Rightarrow (1-\underset{t_{k}}{\max}\left\{\beta_{\sigma (t_{k})}^{3}\right\})\leq \prod_{k=1}^{p}{\left(1-\beta_{\sigma (t_{k})}^{3}\right)}^{\xi_{t_{k}}}\leq (1-\underset{t_{k}}{\min}\left\{\beta_{\sigma (t_{k})}^{3}\right\})$$

$$\Rightarrow \underset{t_{k}}{\min}\left\{\beta_{\sigma (t_{k})}^{3}\right\}\leq 1-\prod_{k=1}^{p}{\left(1-\beta_{\sigma (t_{k})}^{3}\right)}^{\xi_{t_{k}}}\leq \underset{t_{k}}{\max}\left\{\beta_{\sigma (t_{k})}^{3}\right\}$$

$$\Rightarrow \sqrt[{3}]{\underset{t_{k}}{\min}\left\{\beta_{\sigma (t_{k})}^{3}\right\}}\leq \sqrt[{3}]{1-\prod_{k=1}^{p}{\left(1-\beta_{\sigma (t_{k})}^{3}\right)}^{\xi_{t_{k}}}}\leq \sqrt[{3}]{\underset{t_{k}}{\max}\left\{\beta_{\sigma (t_{k})}^{3}\right\}}$$

$$\Rightarrow \underset{t_{k}}{\min}\left\{\beta_{\sigma (t_{k})}\right\}\leq \beta_{t}\leq \underset{t_{k}}{\max}\left\{\beta_{\sigma (t_{k})}\right\}$$ (6)

Comparing inequalities 5 and 6 yields the following result:

$${F_{t}}^{-}\leq FFDOWG(F_{t_{1}}{,F}_{t_{2}},\ldots ,F_{t_{p}})\leq {F_{t}}^{+}.$$

**Theorem 8.** (Monotonicity) Let $F_{t_{k}}=(\alpha_{t_{k}},\beta_{t_{k}})$ and $F_{t_{k}}^{\prime }=(\alpha_{t_{k}}^{\prime },\beta_{t_{k}}^{\prime })$, where k=1,2,…,p, be the sets of FFNs and $\xi_{t}={\left[\xi_{t_{1}},\xi_{t_{2}},\ldots ,\xi_{t_{p}}\right]}^{T}$ be the weight vector corresponding to *t*_*k*_ satisfying $\xi_{t_{k}}\in [0,1]$ and $\sum_{k=1}^{p}\xi_{t_{k}}=1$. In addition, $\left(\sigma (t_{1}),\sigma (t_{2}),\ldots ,\sigma (t_{p})\right)$ denotes the permutation of $t_{k}=t_{1},t_{2},\ldots ,t_{p}$ such that $F_{\sigma (t_{k-1})}\geq F_{\sigma (t_{k})}$, for all *k*. If $\alpha_{\sigma (t_{k})}\leq \alpha_{\sigma (t_{k})}^{\prime }$ and $\beta_{\sigma (t_{k})}\geq \beta_{\sigma (t_{k})}^{\prime }$, then we can establish that:

$$FFDOWG(F_{t_{1}}{,F}_{t_{2}},\ldots ,F_{t_{p}})\leq FFDOWG(F_{t_{1}}^{\prime },F_{t_{2}}^{\prime },\ldots ,F_{t_{p}}^{\prime })$$

**Proof.** The proof of this Theorem follows the same steps as those of Theorem 4.

## 4. Utilization of suggested aggregation operators for addressing MADM challenges

Within this section, we devise an innovative methodology to tackle MADM challenges that incorporate FF data by utilizing FF Dynamic ordered weighted aggregation operators.

- Let {∅_1_, ∅_2_,…,∅_*m*_} represent a set of alternatives.
- Let us consider a set of attributes $\left\{C_{1},C_{2},\ldots ,C_{n}\right\}$, and their associated weight vector *γ* = (*γ*_1_, *γ*_2_,…,*γ*_*n*_)^*T*^, where *γ*_*j*_≥0 for *γ* = 1,2,…,*n* and the sum of all weights *γ*_*j*_ = 1 for all *j*.
- Let *t*_*k*_, where k=1,2,…,p, represent different time periods, each time period is link to a weight vector $\xi_{t}={\left[\xi_{t_{1}},\xi_{t_{2}},\ldots ,\xi_{t_{p}}\right]}^{T}$, with $\xi_{t_{k}}\in [0,1]$ and $\sum_{k=1}^{p}\xi_{t_{k}}=1$.
- Consider the matrix $R_{\left(t_{k}\right)}=[{f_{ij(t_{k})}]}_{m\times n}={\left(\alpha_{ij(t_{k})},\beta_{ij(t_{k})}\right)}_{m\times n}$ representing the FF decision matrices *t*_*k*_ and $\alpha_{ij(t_{k})}$ denotes the extent to which ∅_*i*_ fulfills $C_{j}$ at *t*_*k*_, and $\beta_{ij(t_{k})}$ denotes the extent to which ∅_*i*_ fails to satisfy $C_{j}$ at *t*_*k*_. The aforementioned values adhere to the following condition:

$$\alpha_{ij(t_{k})}\in [0,1],\beta_{ij(t_{k})}\in [0,1]\;and\;{{(\alpha }_{ij(t_{k})})}^{3}+{{(\beta }_{ij(t_{k})})}^{3}\leq 1.$$

In order to address the MADM challenge, the subsequent procedure is devised:

*Step 1*. Obtain FF dynamic decision matrices $R_{\left(t_{k}\right)}=[{f_{ij(t_{k})}]}_{m\times n}={\left(\alpha_{ij(t_{k})},\beta_{ij(t_{k})}\right)}_{m\times n}$ in the form of FFNs *f*_*ij*_ = (*α*_*ij*_, *β*_*ij*_), where *i* varies from 1 to *m* and *j* varies from 1 to *n* for a set of *m* numbers of alternatives and a set of *n* numbers of attributes during the time period *t*_*k*_.

*Step 2*. To derive the permuted FF dynamic decision matrices $R_{\sigma (t_{k})}=[{f_{ij\sigma (t_{k})}]}_{m\times n}=(\alpha_{ij\sigma (t_{k})},\beta_{ij\sigma (t_{k})})$, the subsequent two steps are undertaken:

- Determine the score values of all $C_{j}$, which are associated with each ∅_*i*_, for each $R_{t_{k}}$ throughout the specified time period *t*_*k*_, using Definition 2.
- Determine the FF dynamic permuted decision matrix by arranging the computed values from the above stage of all $C_{j}$, which are associated with each ∅_*i*_, for each $R_{t_{k}}$ during the time period *t*_*k*_ in decreasing sequence.

*Step 3*. This step creates a unified FF decision matrix by combining all of the individual FF dynamic decision matrices represented by $R_{t}=[{f_{ij}]}_{m\times n}={\left(\alpha_{ij},\beta_{ij}\right)}_{m\times n}$, using the developed FFDOW aggregation operators in the following way:

$$•\;FFDOWA(f_{ij\sigma (t_{1})},f_{ij\sigma (t_{2})},\ldots ,f_{ij\sigma (t_{p})})=\left(\sqrt[{3}]{1-\prod_{k=1}^{p}{\left(1-\alpha_{ij\sigma (t_{k})}^{3}\right)}^{\xi_{t_{k}}}},\prod_{k=1}^{p}\beta_{ij\sigma (t_{k})}^{\xi_{t_{k}}}\right)$$

$$•\;FFDOWG(f_{ij\sigma (t_{1})},f_{ij\sigma (t_{2})},\ldots ,f_{ij\sigma (t_{p})})=\left(\prod_{k=1}^{p}\alpha_{ij\sigma (t_{k})}^{\xi_{t_{k}}},\sqrt[{3}]{1-\prod_{k=1}^{p}{\left(1-\beta_{ij\sigma (t_{k})}^{3}\right)}^{\xi_{t_{k}}}}\right)$$

*Step 4*. Normalize (if necessary) the FF decision matrix ℜ_*t*_, using Definition 4 in the following manner:

$$T=\left\{ \begin{array}{c} (\alpha_{ij},\beta_{ij})\;for\;benefit\;criterion\\ (\beta_{ij},\alpha_{ij})\;for\;cost\;criterion \end{array}\right.$$

*Step 5*. Utilize the FF weighted aggregation operators to aggregate all the preference values *f*_*i*_ = (*α*_*i*_, *β*_*i*_) of the alternatives ∅_*i*_, *i* varies from 1 to *m*, obtained from the above step in the following way:

$$•\;FFWA(f_{i1},f_{i2},\ldots ,f_{in})=\left(\sqrt[{3}]{1-\prod_{j=1}^{n}{\left(1-\alpha_{ij}^{3}\right)}^{\gamma_{j}}},\prod_{j=1}^{n}\beta_{ij}^{\gamma_{j}}\right)$$

$$•\;FFWG(f_{i1},f_{i2},\ldots ,f_{in})=\left(\prod_{j=1}^{n}\alpha_{ij}^{\gamma_{j}},\sqrt[{3}]{1-\prod_{j=1}^{n}{\left(1-\beta_{ij}^{3}\right)}^{\gamma_{j}}}\right).$$

 *Step 6*. Compute the score values of each *f*_*i*_ corresponding to each alternative ∅_*i*_ by using Definition 2. If the score values of any pair, such as *f*_*j*_ and *f*_*k*_ are the same, use the accuracy function (Definition 2 (ii)) to rank them.

*Step 7*. Determine the alternative that possesses the highest possible score and assess the resulting ranking.

## 5. An optimal big data analytics tool to efficiently analyze YouTube data under dynamic Fermatean fuzzy environment

In this section, we utilized the suggested approaches to choose the most effective big data analytics tool for the analysis of YouTube data.

### 5.1. Big data

The advancement of technology produces various types of structured data, the predominant type comprises semi-structured or unstructured data, in the form of big data. The term big data refers to vast and complex data collections that require the use of advanced and affordable evaluation and analysis tools in order to extract insights and facilitate decision-making [39]. Only 5% of the overall data landscape consists of structured data, which includes forms such as spreadsheets or relational databases [51]. Unstructured data, encompassing images, online text, video and audio that do not have certain organization and require specialized analytics or tools for analysis [51]. In 2001, Laney initially formulated the notion of big data by delineating its characteristics in relation to Volume, Velocity, and Variety. In the era of information, decision makers are presented with enormous amounts of data [52]. The term big data refers to datasets that are not only large in size but also have a high in variety and velocity, making them difficult to manage using traditional tools and methodologies. Big data, has emerged as a powerful resource. Big data is widely used in several fields such as social networks, healthcare, academics, transit planning, aerospace, gas and oil development, e-commerce, finance, insurance, telecommunications, surveillance, military, and many more industries [53]. However, the significant collection of data becomes valuable only when skillfully utilized to express relevant stories. Data analytics is a crucial tool that helps to clearly and easily communicate data stories.

### 5.2. Social media

Big Data has become increasingly prevalent in modern-day, as businesses and organizations engage in passionate discussions regarding their Big Data solutions and analytical applications. Although the sources of the data employed in these applications are varied, social media data comes out as an especially compelling category for the majority of organizations. A considerable proportion of the worldwide population utilizes social media platforms. According to the reputable social monitoring platform Brandwatch, as of May 2019 [54], more than 3.4 billion people globally were engaged in active use of social media sites. A substantial volume of structured, semi-structured, and unstructured data is generated expeditiously within this social media-based platform [55]. The capability for instantaneous connection and communication with individuals and organizations across vast distances constitutes a pivotal facet of contemporary society. Social Media applications facilitate swift information dissemination, the exchange of comments, opinions, ideas, and media content encompassing images, text, video and audio sharing. Social Media applications enable individual users to engage in simultaneous communication with a considerable number of other users. The implementation of data mining and analysis methodologies enables the prediction of particular user actions within these applications. At this time, a multitude of technologies play a crucial role in the gathering, examination, and presentation of this data. By utilizing these technologies, individuals are able to tackle a wide range of challenges in numerous industries, such as finance, advertising, medicine, education and environmental science.

The increasing volume of data produced by platforms based on social media has rekindled interest in the realm of big data analytics. The primary objective of this endeavor is to extract significant insights from the extensive array of content that billions of users’ daily share, which includes images, text, video, audio, GIFs, blogs and more [51]. Organizations are dedicating substantial resources to make substantial investments in big data analytics, specifically to analyze online behaviors, with a particular focus on social networking platforms including Facebook, YouTube, Instagram, Twitter, TikTok, WhatsApp, LinkedIn, and numerous blogs [53]. Numerous organizations, through their analysts, are dedicating substantial time, financial resources, and efforts to extract valuable insights from the vast volumes of social data. The utilization of efficient methodologies and analytical tools is crucial when assessing the continuously increasing volumes of data originating from various social media platforms. The investigation of social media has experienced a substantial upswing in recent years, accompanied by the development of several big data analysis models for an in-depth exploration of social data [55]. Social media engagement is widely recognized as a prominent online activity that has achieved a remarkable degree of global exposure. The global count of active social media users has accumulated to 4.48 billion individuals as of 2021. By 2022, social media users topped 4.59 billion. This number reached to 4.95 billion in October 2023, 61.4 percent of the world population.

Big data has evolved from a trend to a need. Large companies like Google, Amazon, Microsoft, and Facebook have invested heavily in big data in recent years. These investments boost expertise, efficiency, and strategic approaches. The efficacy of a product is now predominantly contingent on public response. Decades ago, companies exclusively relied on television as a medium for product promotion. Likewise, in the past, filmmakers would exclusively employ television media for the promotion of their films and songs. However, in our contemporary, more technologically advanced and digitalized era, companies have transitioned to leveraging platforms such as YouTube for marketing and brand promotion. This involves uploading product advertisement videos to YouTube and movie makers utilize the YouTube for songs and movie trailers. The reception of both products and movies by the public is gauged through quantitative metrics such as the number of views, likes and comments garnered on the respective videos.

### 5.3. YouTube

YouTube is widely recognized as the preeminent video-sharing platform on a global scale. A significant statistic indicates that mobile devices account for over 70% of YouTube views [56]. YouTube is renowned for its extensive range of content, which originates from a diverse array of viewers, is conveyed in multiple languages, and encompasses an array of genres [57]. Videos are among the most engaging forms of media, and the significant level of interaction on this social media platform emphasizes the need for thorough examination of video content and related comments. YouTube has over 2.70 billion monthly active users as of 2023. In excess of one-fourth of the global population engages with YouTube on a monthly basis, and approximately half of the worldwide internet users have accessibility to YouTube. The growth of YouTube users has exhibited remarkable expansion over the last decade, escalating from 0.8 billion in 2012 to an impressive 2.70 billion in 2023. Within recent years, YouTube has transcended its initial status, evolving into a global broadcasting platform and assuming a pivotal role within the mainstream media landscape. Currently, YouTube has established collaborations with prominent media outlets that cover a wide range of channels, including news, entertainment, and gaming. The integration of high-definition videos into the platform has enabled the widespread distribution of content produced by a variety of niche creators, including vloggers and gamers, as well as individuals who contribute high-resolution material.

Nowadays, YouTube holds a significant impact on internet traffic, however, it faces a notable challenge in terms of scalability. The effective analysis, storage, and processing of such massive amounts of data within condensed time periods present an exceptionally challenging endeavor. Majority of the data derived from the billions of YouTube videos is unstructured. Mastering the rapid, effective, and accurate examination of this unstructured or semi-structured data continues to be a significant obstacle. Google statistics indicate that YouTube has more than a billion users, which accounts for nearly one-third of the global Internet population. On a daily basis, these users consume one billion hours of video content, which generates billions of views [58]. YouTube gathers a diverse array of conventional data metrics encompassing views, votes, likes comments, and video duration. The aggregation of these specified data points constitutes a compelling dataset for analysis, offering implicit insights into user behavior, video content, categories, and community interests. Movie production houses unveil promotional content and songs on YouTube, corporate brands deploy advertisements on the platform for promotional purposes, and emerging artists showcase and publicize their work for wider exposure. These instances represent few examples. The success metrics for movies, songs, brand advertisements, and artists predominantly hinge on the metrics of viewership, likes, and comments. Notably, companies and artists can not only analyze their individual performance but also conduct comparative analyses with their competitors.

#### 5.3.1. YouTube analytics

YouTube users who create and upload videos on their YouTube channels are frequently curious about the geographical whereabouts of those who view their content, which videos are generating the most interest, and who is viewing their videos. YouTube Analytics functions as a resilient platform, providing an all-encompassing interface that compiles this data with an extensive array of metrics. Similar to the Google Analytics, YouTube Analytics provides vital insights into the efficacy of videos hosted on the platform. The metrics obtained from YouTube video analytics are crucial in improving the understanding of how videos are performed. With respect to organizations contending with fiercely competitive environments, YouTube Analytics provides crucial insights for users to gain a deeper comprehension of the reach and visibility of their content. YouTube Analytics can be employed to monitor objectives such as content engagement and viewership. YouTube Analytics dashboards feature, among other things, real-time video statistics. YouTube, being the second-largest search engine subsequent to Google, attracts millions of visitors in quest of pertinent video content.

#### 5.3.2. YouTube data collection and analysis tools

Data collection and analysis tools are utilized by content creators on YouTube for the following purposes: to comprehend the demographics of their audience, optimize content, refine monetization strategies, monitor performance metrics, conduct competitive analyses, interact with viewers, and enhance SEO. The YouTube SEO process optimizes channels, categories, and videos. To increase views, these include incorporating popular keywords into video titles and descriptions and utilizing hashtags. By increasing a website’s ranking in search engines, SEO boosts website traffic. Four applications for YouTube data capture and analysis are described briefly in the following section.

*a*. *VidIQ*. VidIQ is an internet-based educational platform that provides video courses and data analysis on the expansion of YouTube channels. The website offers a Google Chrome extension that enables users to examine YouTube analytics data. VidIQ and TubeBuddy are frequently compared because to their comparable characteristics. It offers valuable information that enhance the discoverability of videos. The video SEO optimization tool greatly assists in improving titles, tags, and descriptions, while the channel audit function provides helpful suggestions for enhancing channel development. VidIQ provides a browser extension that is easy to use.

*b*. *TubeBuddy*. TubeBuddy emerges as a prominent tool within the YouTube ecosystem, providing an array of features designed to aid creators in the efficient management and optimization of their channels. Functioning as a robust YouTube SEO tool, competitor analysis resource, and a time-saving Chrome extension tailored for video creators, TubeBuddy exhibits the capability to find related keywords and tags conducive to enhancing video visibility. The utilization of TubeBuddy is instrumental in enhancing search engine visibility for video content, thereby contributing to the optimization and expansion of the YouTube channel.

*c*. *Unbox social*. Unbox Social is a comprehensive and well-designed solution that provides social media managers and agencies with a wide range of metrics and statistics for four major social media platforms: YouTube, Instagram, Facebook, and Twitter. Unbox Social stands out due to its exceptional reporting capabilities. The social reports produced by Unbox Social feature informative graphs that provide a visually intuitive depiction of crucial social media data, hence improving the accessibility and understanding of essential insight.

*d*. *Quintly*. Quintly is a valuable tool offering insights into the performance of your YouTube content. Through intuitive dashboard visualization, users can effortlessly comprehend the YouTube metrics relevant to their objectives. Quintly serves as a comprehensive social media analytics platform, affording users the capability to monitor and analyze both their own social media channels and those of competitors within a unified interface. Quintly’s competitor analysis feature empowers users to assess their social media performance relative to competitors, offering valuable insights into competitors’ activities and highlighting areas for potential enhancement in one’s social media strategy.

### 5.4. Illustration

A YouTuber wants to optimize and manage his tech review channel using big data analytics tool. In order to accomplish this, he establishes a methodical process for assessing and choosing the most effective big data analytics tool alternatives for YouTube data to enhance his content strategy, maximize viewership and boost engagement. His study focuses on employing big data analytics tools to analyze YouTube data within the FF environment. In order to fulfill his requirements, he engaged professionals who are experts in data science, machine learning, and information technology. These experts are responsible for utilizing advanced analytics to extract valuable information from the extensive and dynamic platform of YouTube. These professionals have been assigned with big data analytics tools for analyzing YouTube data using number of alternative analytics tools and attributes that are crucial for analyzing YouTube data.

Let {∅_1_, ∅_2_, ∅_3_, ∅_4_}, represents set of Big data analytics tool alternatives, where

- ∅_1_: Quintly
- ∅_2_: Unbox Social
- ∅_3_: TubeBuddy
- ∅_4_: VidIQ

Moreover, $\left\{C_{1},C_{2},C_{3},C_{4}\right\}$ represents set of pertinent attributes, where

- $C_{1}:$ Performance tracking
- $C_{2}:$ Competitor tracking analytics
- $C_{3}:$ Audience engagement metrics (likes, comments, shares)
- $C_{4}:$ Search Engine Optimization (SEO)

The decision-maker assesses the four feasible alternatives ∅_1_, ∅_2_, ∅_3_ and ∅_4_ by applying FF information to the attributes $C_{1},C_{2},C_{3}$ and $C_{4}$ at three distinct time intervals *t*_1_, *t*_2_, and *t*_3_. In this context, *t*_1_, *t*_2_, and *t*_3_ correspond to the years 2021, 2022, and 2023, respectively. The decision-maker has allocated weight vector *ξ*_*t*_ = [0.2, 0.35, 0.45]^*T*^ to these time periods, and weight vector *γ* = (0.1, 0.2, 0.3, 0.4)^*T*^ to the attributes.

The expert opinions for evaluating the dependability of the alternatives ∅_1_, ∅_2_, ∅_3_ and ∅_4_ that are linked to $C_{1},C_{2},C_{3}$ and $C_{4}$ during the designated time intervals *t*_1_, *t*_2_, and *t*_3_ can be found in the FF decision matrices in Tables 1–3 respectively.

**Table 1:** FF decision matrix $R_{t_{1}}$.

	$C_{1}$	$C_{2}$	$C_{3}$	$C_{4}$
∅_1_	(0.8,0.6)	(0.8,0.3)	(0.6,0.4)	(0.8,0.5)
∅_2_	(0.7,0.4)	(0.6,0.7)	(0.8,0.3)	(0.7,0.8)
∅_3_	(0.9,0.4) (0.9,0.4)	(0.7,0.6)	(0.7,0.3)	(0.6,0.3)
∅_4_	(0.8,0.6)	(0.8,0.5)	(0.9,0.6)	(0.8,0.4)

**Table 2:** FF decision matrix $R_{t_{2}}$.

	$C_{1}$	$C_{2}$	$C_{3}$	$C_{4}$
∅_1_	(0.8,0.5)	(0.8,0.3)	(0.6,0.4)	(0.6,0.5)
∅_2_	(0.7,0.3)	(0.7,0.6)	(0.7,0.5)	(0.6,0.7)
∅_3_	(0.9,0.4)	(0.8,0.5)	(0.7,0.2)	(0.8,0.4)
∅_4_	(0.7,0.5)	(0.8,0.4)	(0.8,0.5)	(0.7,0.5)

**Table 3:** FF decision matrix $R_{t_{3}}$.

	$C_{1}$	$C_{2}$	$C_{3}$	$C_{4}$
∅_1_	(0.9,0.6)	(0.7,0.2)	(0.7,0.5)	(0.8,0.5)
∅_2_	(0.8,0.3)	(0.7,0.5)	(0.8,0.6)	(0.8,0.7)
∅_3_	(0.7,0.4)	(0.8,0.5)	(0.9,0.4)	(0.9,0.4)
∅_4_	(0.8,0.7)	(0.9,0.4)	(0.9,0.6)	(0.9,0.3)

The given MADM scenario is investigated using the FFDOWA and FFDOWG operators, as follows:

*Step 1*. The permuted FF decision matrix $R_{\sigma (t_{1})}=[{f_{\left(ij)\sigma (t_{1}\right)}]}_{4\times 4}=(\alpha_{\left(ij)\sigma (t_{1}\right)},\beta_{\left(ij)\sigma (t_{1}\right)})$, is determined as follows:

- Determine the scores of all four attributes for their respective alternatives in matrix $R_{t_{1}}$ during the time period *t*_1_ using Definition 2:
  - For alternative ∅_**1**_**,** we have
$$S(f_{11})=0.296,\;S(f_{12})=0.485,\;S(f_{13})=0.152\;and\;S(f_{14})=0.387$$
  - For alternative ∅_**2**_**,** we have
$$S(f_{21})=0.279,\;S(f_{22})=-0.127,\;S(f_{23})=0.485\;and\;S(f_{24})=-0.169$$
  - For alternative ∅_**3**_**,** we have
$$S(f_{31})=0.665,\;S(f_{32})=0.127,\;S(f_{33})=0.316\;and\;S(f_{34})=0.189$$
  - For alternative ∅_**4**_**,** we have
$$S(f_{41})=0.296,\;S(f_{42})=0.387,\;S(f_{43})=0.513\;and\;S(f_{44})=0.448$$
- Sort the acquired values from the previous step in decreasing order for each alternative in the following way:
  - For alternative ∅_**1**_**,** we have
$$S(f_{12})≻S(f_{14})≻S(f_{11})≻S(f_{13})$$
  - For alternative ∅_**2**_, we have
$$S(f_{23})≻S(f_{21})≻S(f_{22})≻S(f_{24})$$
  - For alternative ∅_**3**_, we have
$$S(f_{31})≻S(f_{33})≻S(f_{34})≻S(f_{32})$$
  - For alternative ∅_**4**_, we have
$$S(f_{43})≻S(f_{44})≻S(f_{42})≻S(f_{41})$$

The permuted FF decision matrix $R_{\sigma (t_{2})}=[{f_{\left(ij)\sigma (t_{2}\right)}]}_{4\times 4}=(\alpha_{\left(ij)\sigma (t_{2}\right)},\beta_{\left(ij)\sigma (t_{2}\right)})$, is determined as follows:

- Determine the scores of all four attributes for their respective alternatives in matrix $R_{t_{2}}$ during the time period *t*_2_ using Definition 2:
  - For alternative ∅_**1**_, we have
$$S(f_{11})=0.387,\;S(f_{12})=0.485,\;S(f_{13})=0.152\;and\;S(f_{14})=0.091$$
  - For alternative ∅_**2**_**,** we have
$$S(f_{21})=0.316,\;S(f_{22})=0.127,\;S(f_{23})=0.218\;and\;S(f_{24})=-0.127$$
  - For alternative ∅_**3**_**,** we have
$$S(f_{31})=0.665,\;S(f_{32})=0.387,\;S(f_{33})=0.335\;and\;S(f_{34})=0.448$$
  - For alternative ∅_**4**_**,** we have
$$S(f_{41})=0.218,\;S(f_{42})=0.448,\;S(f_{43})=0.387\;and\;S(f_{44})=0.218$$
- Arrange the obtained values from the above stage corresponding to each alternative in descending order as follows:
  - For alternative ∅_**1**_, we have
$$S(f_{12})≻S(f_{11})≻S(f_{13})≻S(f_{14})$$
  - For alternative∅_**2**_, we have
$$S(f_{21})≻S(f_{23})≻S(f_{22})≻S(f_{24})$$
  - For alternative ∅_**3**_, we have
$$S(f_{31})≻S(f_{34})≻S(f_{32})≻S(f_{33})$$
  - For alternative ∅_**4**_, we have
$$S(f_{42})≻S(f_{43})≻S(f_{41})≻S(f_{44})$$

The permuted FF decision matrix $R_{\sigma (t_{3})}=[{f_{\left(ij)\sigma (t_{3}\right)}]}_{4\times 4}=(\alpha_{\left(ij)\sigma (t_{3}\right)},\beta_{\left(ij)\sigma (t_{3}\right)})$, is determined as follows:

- Determine the scores of all four attributes for their respective alternatives in matrix $R_{t_{3}}$ during the time period *t*_3_ using Definition 2:
  - For alternative ∅_**1**_, we have
$$S(f_{11})=0.513,\;S(f_{12})=0.335,\;S(f_{13})=0.218\;and\;S(f_{14})=0.387$$
  - For alternative ∅_**2**_**,** we have
$$S(f_{21})=0.485,\;S(f_{22})=0.218,\;S(f_{23})=0.296\;and\;S(f_{24})=0.169$$
  - For alternative ∅_**3**_**,** we have
$$S(f_{31})=0.279,\;S(f_{32})=0.387,\;S(f_{33})=0.665\;and\;S(f_{34})=0.665$$
  - For alternative ∅_**4**_**,** we have
$$S(f_{41})=0.169,\;S(f_{42})=0.665,\;S(f_{43})=0.513\;and\;S(f_{44})=0.702$$
- Arrange the obtained values from the above stage corresponding to each alternative in descending order as follows:
  - For alternative ∅_**1**_**,** we have
$$S(f_{11})≻S(f_{14})≻S(f_{12})≻S(f_{14})$$
  - For alternative ∅_**2**_**,** we have
$$S(f_{21})≻S(f_{23})≻S(f_{22})≻S(f_{24})$$
  - For alternative ∅_**3**_**,** we have
$$S(f_{33})≻S(f_{34})≻S(f_{32})≻S(f_{31})$$
  - For alternative ∅_**4**_**,** we have
$$S(f_{44})≻S(f_{42})≻S(f_{43})≻S(f_{41})$$

**Step 2.** Formulate the permuted FF decision matrices $R_{\sigma (t_{k})}=[{f_{\left(ij)\sigma (t_{k}\right)}]}_{4\times 4}=(\alpha_{\left(ij)\sigma (t_{k}\right)},\beta_{\left(ij)\sigma (t_{k}\right)})$, where *t* = 1,2,3 in the framework of the information obtained from step 1. The matrices are displayed in Tables 4–6.

**Table 4:** Permuted FF decision matrix $R_{\sigma (t_{1})}$.

	$C_{1}$	$C_{2}$	$C_{3}$	$C_{4}$
∅_1_	(0.8,0.3)	(0.8,0.5)	(0.8,0.6)	(0.6,0.4)
∅_2_	(0.8,0.3)	(0.7,0.4)	(0.6,0.7)	(0.7,0.8)
∅_3_	(0.9,0.4)	(0.7,0.3)	(0.6,0.3)	(0.7,0.6)
∅_4_	(0.9,0.6)	(0.8,0.4)	(0.8,0.5)	(0.8,0.6)

**Table 5:** Permuted FF decision matrix $R_{\sigma (t_{2})}$.

	$C_{1}$	$C_{2}$	$C_{3}$	$C_{4}$
∅_1_	(0.8,0.3)	(0.8,0.5)	(0.6,0.4)	(0.6,0.5)
∅_2_	(0.7,0.3)	(0.7,0.5)	(0.7,0.6)	(0.6,0.7)
∅_3_	(0.9,0.4)	(0.8,0.4)	(0.8,0.5)	(0.7,0.2)
∅_4_	(0.8,0.4)	(0.8,0.5)	(0.7,0.5)	(0.7,0.5)

**Table 6:** Permuted FF decision matrix $R_{\sigma (t_{3})}$.

	$C_{1}$	$C_{2}$	$C_{3}$	$C_{4}$
∅_1_	(0.9,0.6)	(0.8,0.5)	(0.7,0.2)	(0.7,0.5)
∅_2_	(0.8,0.3)	(0.8,0.6)	(0.7,0.5)	(0.8,0.7)
∅_3_	(0.9,0.4)	(0.9,0.4)	(0.8,0.5)	(0.7,0.4)
∅_4_	(0.9,0.3)	(0.9,0.4)	(0.9,0.6)	(0.8,0.7)

**Step 3.** By employing the FFDOWA operator, every permuted FF decision matrix is merged into a single FF decision matrix denoted as ℜ_*t*_. Table 7 lists these FFNs.

**Table 7:** Unified FF decision matrix obtained by applying FFDOWA operator.

	$C_{1}$	$C_{2}$	$C_{3}$	$C_{4}$
∅_1_	(0.855,0.409)	(0.8,0.5)	(0.698,0.317)	(0.651,0.478)
∅_2_	(0.771,0.3)	(0.752,0.519)	(0.683,0.570)	(0.729,0.718)
∅_3_	(0.9,0.4)	(0.844,0.377)	(0.773,0.451)	(0.7,0.340)
∅_4_	(0.873,0.381)	(0.855,0.432)	(0.836,0.542)	(0.771,0.603)

Similarly, by employing the FFDOWG operator, every permuted FF decision matrix is merged into a single FF decision matrix denoted as ℜ_*t*_. Table 8 lists these FFNs.

**Table 8:** Unified FF decision matrix obtained by applying FFDOWG operator.

	$C_{1}$	$C_{2}$	$C_{3}$	$C_{4}$
∅_1_	(0.843,0.489)	(0.8,0.5)	(0.681,0.417)	(0.643,0.483)
∅_2_	(0.763,0.3)	(0.743,0.538)	(0.678,0.589)	(0.704,0.724)
∅_3_	(0.9,0.4)	(0.821,0.384)	(0.755,0.473)	(0.7,0.427)
∅_4_	(0.863,0.432)	(0.843,0.441)	(0.805,0.550)	(0.763,0.627)

**Step 5.** Utilize the FFWA operator to obtain the accumulated values *f*_1_, *f*_2_, *f*_3_, and *f*_4_ of all four alternatives. The detail is summarized in Table 9.

**Table 9:** Aggregated values of alternatives using FFWA operator.

Alternatives	*f*_*i*_
∅_1_	(0.731,0.419)
∅_2_	(0.726,0.575)
∅_3_	(0.787,0.383)
∅_4_	(0.822,0.521)

In a similar way, utilize the FFWG operator to obtain the accumulated values *f*_1_, *f*_2_, *f*_3_, and *f*_4_ of all four alternatives. The detail is summarized in Table 10.

**Table 10:** Aggregated values of alternatives using FFWG operator.

	*f*_*i*_
∅_1_	(0.702,0.469)
∅_2_	(0.709,0.635)
∅_3_	(0.758,0.432)
∅_4_	(0.800,0.560)

**Step 5.** Utilizing Definition 2, compute the score values of the overall FF preferences *f*_1_, *f*_2_, *f*_3_, and *f*_4_, obtained in the framework of FFDOWA operator in order to rank all the alternatives.


$$S(f_{1})=0.317\;S(f_{2})=0.192$$

$$S(f_{3})=0.431\;S(f_{4})=0.413$$

Similarly, Utilizing Definition 2, compute the score values of the overall FF preferences *f*_1_, *f*_2_, *f*_3_, and *f*_4_, obtained in the framework of FFDOWG operator in order to rank all the alternatives.


$$S(f_{1})=0.242\;S(f_{2})=0.100$$

$$S(f_{3})=0.354\;S(f_{4})=0.336$$

**Step 6.** This leads us to the following ranking of all alternatives within the framework of FFDOWA and FFDOWG operators: ∅_3_≻∅_4_≻∅_1_≻∅_2_. Therefore, TubeBuddy emerges as the optimal alternative for analyzing YouTube data.

**Fig 1:**
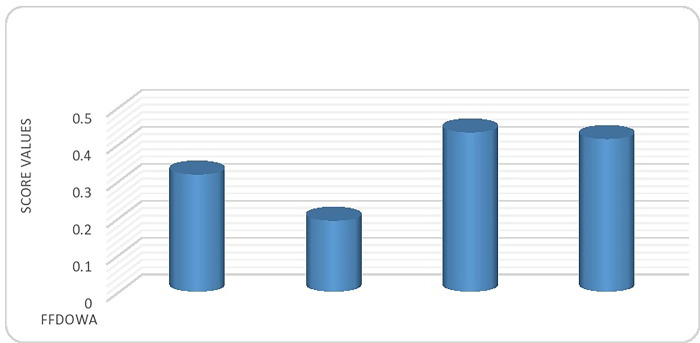
Ranking of alternatives using FFDOWA.

**Fig 2:**
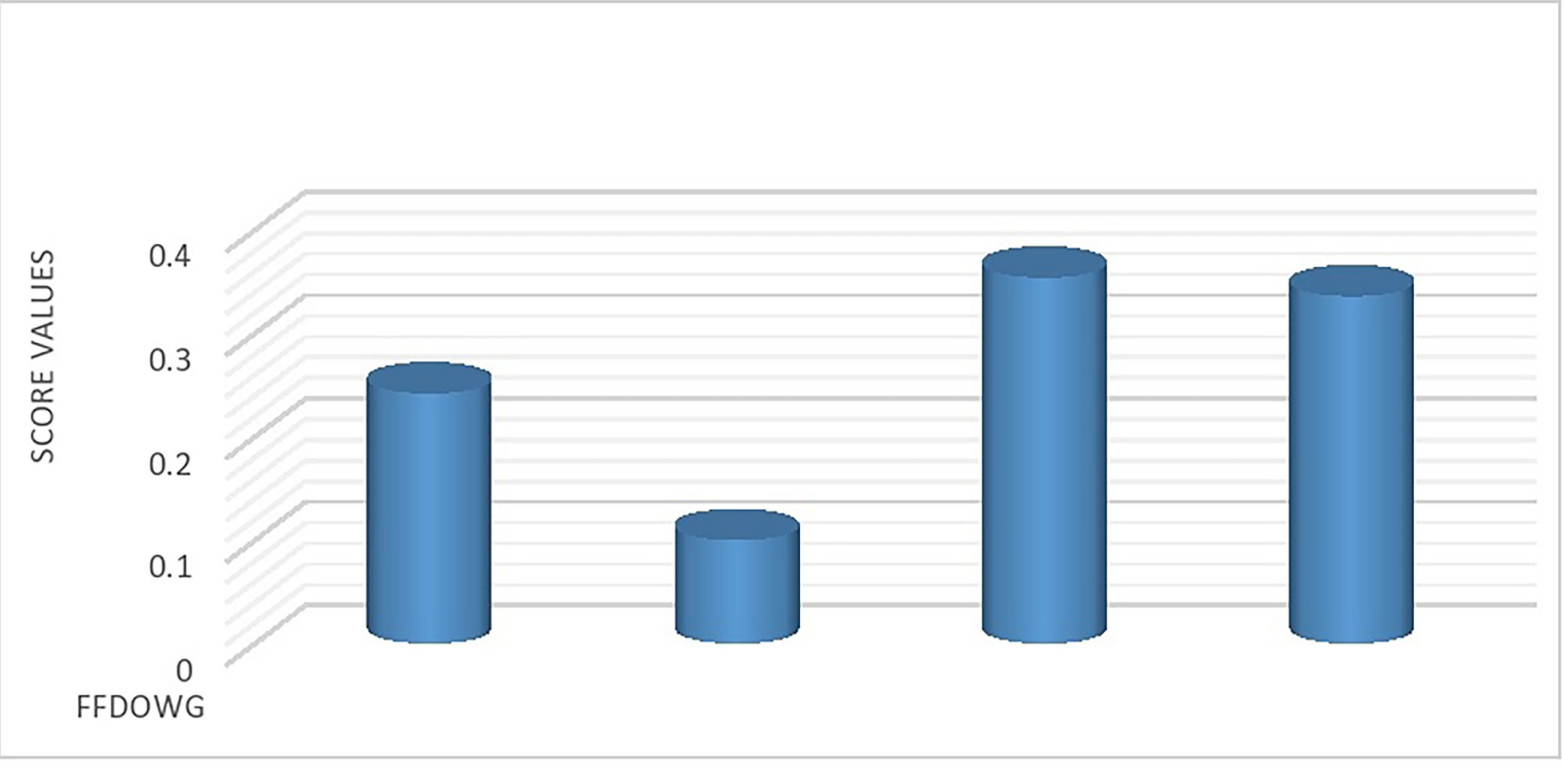
Ranking of alternatives using FFDOWG.

Figs 1 and 2 visually display the score ratings for the alternatives obtained by the FFDOWA and FFDOWG operators respectively.

### 5.5. Comparative analysis

In the following section, we analyze the MADM problem discussed earlier by assessing the efficacy and reliability of our proposed operators in comparison to operators in the IF and FF dynamic settings. To collect and consolidate identical data, we employ a variety of methodologies, including the IFDWG, FFDWA, IFDWA, and FFDWG operators. Table 11 contains a compilation of the outcomes generated by employing these operators, while Table 12 presents the outcomes produced based on their ranking.

**Table 11:** Accumulated values of alternatives acquired from various established operators.

	IFDWA [46]	IFDWG [46]	FFDWA [49]	FFDWG [49]
$f(A_{1})$	(0.737,0.424)	(0.714,0.449)	(0.730,0.424)	(0.714,0.465)
$f(A_{2})$	(0.733,0.563)	(0.719,0.611)	(0.734,0.563)	(0.719,0.630)
$f(A_{3})$	(0.818,0.375)	(0.789,0.395)	(0.795,0.375)	(0.789,0.411)
$f(A_{4})$	(0.843,0.455)	(0.825,0.482)	(0.843,0.455)	(0.825,0.500)

**Table 12:** Ranking of alternatives based on score values.

Methods	*S*(*f*_1_)	*S*(*f*_2_)	*S*(*f*_3_)	*S*(*f*_4_)	Ranking Order
IFDWA [46]	0.313	0.170	0.443	0.388	∅_3_≻∅_4_≻∅_1_≻∅_2_
IFDWG [46]	0.265	0.108	0.394	0.343	∅_3_≻∅_4_≻∅_1_≻∅_2_
FFDWA [49]	0.312	0.216	0.449	0.504	∅_4_≻∅_3_≻∅_1_≻∅_2_
FFDWG [49]	0.263	0.121	0.421	0.436	∅_4_≻∅_3_≻∅_1_≻∅_2_
FFDOWA	0.317	0.192	0.431	0.413	∅_3_≻∅_4_≻∅_1_≻∅_2_
FFDOWG	0.242	0.100	0.354	0.336	∅_3_≻∅_4_≻∅_1_≻∅_2_

This demonstrates that our proposed methodologies are authentic and applicable for MADM problems. The main logic behind the superiority of our proposed approaches is that the FFS possesses a broader structure that represents an extension of IFS as it fulfills the constraint *α*^3^+*β*^3^≤1. As a result, it is more adaptable to dealing with ambiguity in decision-making. It is evident that the techniques outlined in [46] can be considered a particular instance of the novel approaches introduced in this current study. Moreover, the methodologies developed in dynamic IF knowledge [46, 47] have limited applicability compared to the strategies presented in this study. This is due to the fact that the set of Fermatean membership degrees is more efficient than the set of intuitionistic membership degrees. So, dynamic FFS offers a broader selection of options for recognizing and addressing uncertainty than dynamic IFS.

The mathematical frameworks proposed for the operators, which are constructed using FFSs with time periods, exhibit exceptional efficacy and comprehensiveness. This illustrates the dominance of the suggested methodology compared to other approaches in the literature.

**Fig 3:**
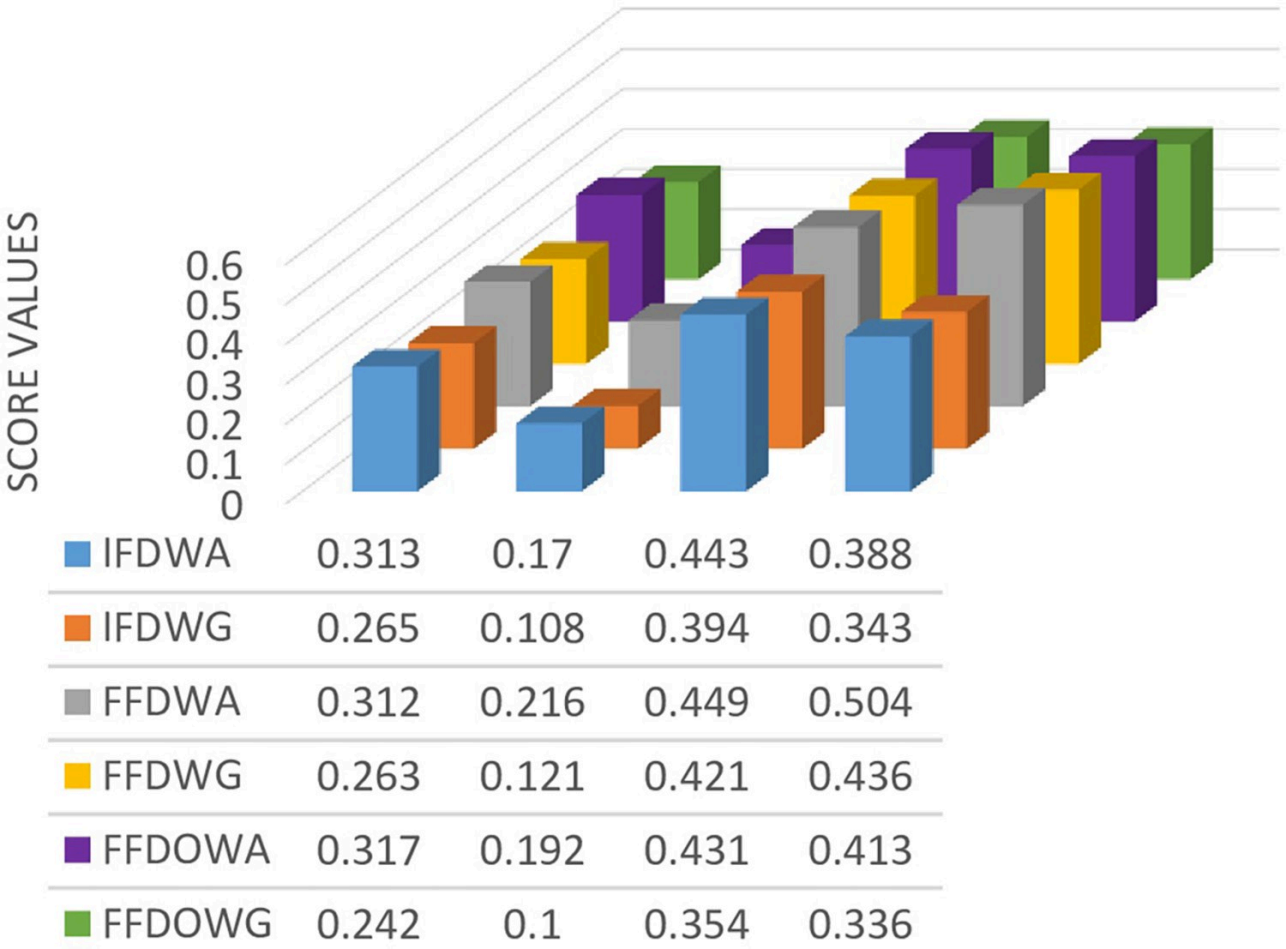
Ranking of alternatives using different operators.

Fig 3 visually displays the comparison of score ratings for the alternatives obtained by newly defined operators and already existing operators.

**Remark 1.** The collection and analysis of YouTube data in this study adhered to the terms and conditions specified by the source of the data.

## 6. Conclusions

The purpose of this research is to present novel approaches to resolving decision-making challenges in a dynamic FF environment. The extant literature has documented a multitude of operators that have demonstrated advantageous outcomes. Nevertheless, it is critical to acknowledge that in the extant literature, none of these operators have specifically considered the temporal aspect of the FF setting. Therefore, utilizing a dynamic FF model is a more effective approach for representing data pertaining to time-varying situations, as it can more efficiently manage two-dimensional data within a cohesive framework. Based on these variables, we have introduced a new collection of operators called FFDOWA and FFDOWG. We have also analyzed many characteristics of these operators. Moreover, we have presented a mathematical mechanism to tackle dynamic FF MADM problems using newly define strategies. Additionally, we have provided an illustration of how to effectively apply these newly developed strategies to choose the most efficient big data analytics tool for analyzing YouTube data. Finally, a comparative analysis is conducted to underscore the significance and dependability of these innovative methodologies in comparison to recently available strategies.

### 6.1. Limitations of the current study

The suggested approaches improve reliability and outer performance compared to already existing strategies; however, these techniques have the following limitations:

- These strategies are restricted to accepting only two parameters, making them unsuitable for model cases that involve more than two parameters, for instance, picture fuzzy information and spherical fuzzy information.
- FFSs do not account for scenarios in which the cubic sum of degrees of membership and non-membership is greater than 1.

### 6.2. Future directions

Our primary focus of future work will be to rectify the limitations of the current study by defining these strategies to more generalize environments, for instance, within the context of interval-valued Fermatean fuzzy sets, bipolar fuzzy sets, spherical fuzzy, picture fuzzy, complex Fermatean fuzzy settings, quasirung fuzzy sets [59], and 3, 4-quasirung fuzzy sets [60]. Furthermore, this study will broaden its scope to address a variety of decision-making challenges across multiple domains, with the aim of optimizing MADM. These domains include identifying the most suitable big data analytic tool for analyzing data from Facebook, Instagram, or Twitter, determining medical treatments, dynamic financial strategies, selecting harvesting strategies for ecological populations, forecasting economic trends, and selecting solar panels.
