# Expression of Concern: Selecting the foremost big data tool to optimize YouTube data in dynamic Fermatean fuzzy knowledge

**DOI:** 10.1371/journal.pone.0355527

**Published:** 2026-08-12

**Authors:** The PLOS One Editors

After this article [1] was published, the following concerns were noted:

- The articles cited as References 42 and 43 were retracted after [1] was published.
- The articles cited as References 53 and 54 do not appear to support their respective corresponding cited statements.

In addition, the *PLOS One* Editors have concerns about the article’s peer review and compliance with the PLOS Authorship policy.

The corresponding author acknowledged the concerns about References 53 and 54 and provided an explanation for their inclusion in the article; however, the Editors do not consider this response to have resolved the concerns.

In light of the cumulative issues, the *PLOS One* Editors issue this Expression of Concern. Readers are advised to interpret the article [1] with caution.

## References

[1] Alghazzawi D, Razaq A, Alolaiyan H, Noor A, Khalifa HAE-W, Xin Q. Selecting the foremost big data tool to optimize YouTube data in dynamic Fermatean fuzzy knowledge. PLoS One. 2024;19(8):e0307381. doi: 10.1371/journal.pone.0307381 39178296 PMC11343475

